# Habitat Association Predicts Population Connectivity and Persistence in Flightless Beetles: A Population Genomics Approach Within a Dynamic Archipelago

**DOI:** 10.1111/mec.17577

**Published:** 2024-11-05

**Authors:** Emmanouil Meramveliotakis, Joaquín Ortego, Ioannis Anastasiou, Alfried P. Vogler, Anna Papadopoulou

**Affiliations:** ^1^ Department of Biological Sciences, Faculty of Pure and Applied Sciences University of Cyprus Nicosia Cyprus; ^2^ Department of Ecology and Evolution Estación Biológica de Doñana, EBD‐CSIC Seville Spain; ^3^ Department of Biology, School of Science National and Kapodistrian University of Athens Athens Greece; ^4^ Department of Life Sciences Natural History Museum London UK; ^5^ Department of Life Sciences, Faculty of Natural Sciences, Silwood Park Campus Imperial College London Ascot UK

**Keywords:** Aegean, ddRAD, demography, extinction–recolonisation, habitat stability, sand dunes

## Abstract

Habitat association has been proposed to affect evolutionary dynamics through its control on dispersal propensity, which is considered a key trait for lineage survival in habitats of low durational stability. The Habitat Constraint hypothesis predicts different micro‐ and macroevolutionary patterns for stable versus dynamic habitat specialists, but the empirical evidence remains controversial and in insects mostly derives from winged lineages. We here use genome‐wide SNP data to assess the effect of habitat association on the population dynamics of two closely related flightless lineages of the genus *Eutagenia* (Coleoptera: Tenebrionidae), which are co‐distributed across the Cyclades islands in the Eastern Mediterranean but are associated with habitat types of different presumed stability: the psammophilous lineage is associated with dynamic sandy coastal habitats, while the geophilous lineage is associated with comparatively stable compact soil habitats. Our comparative population genomic and demographic analyses support higher inter‐island gene flow in the psammophilous lineage, presumably due to the physical properties of dynamic sand‐dune habitats that promote passive dispersal. We also find consistent bottlenecks in the psammophilous demes, suggesting that lineage evolution in the dynamic habitat is punctuated by local extinction and recolonisation events. The inferred demographic processes are surprisingly uniform among psammophilous demes, but vary considerably among geophilous demes depending on historical island connectivity, indicating more stringent constraints on the dynamic habitat lineage. This study extends the Habitat Constraint hypothesis by demonstrating that selection on dispersal traits is not the only mechanism that can drive consistent differences in evolutionary dynamics between stable versus dynamic habitat specialists.

## 1 Introduction

Habitats can be considered as ‘templets’ (Greenslade 1983; Southwood 1977, 1988) that encompass a set of environmental factors, constraining the evolution of organismal traits and life histories. A major component of the ‘habitat templet’ concept is the habitat durational stability, which is a key factor in determining certain ecological strategies, especially with regard to dispersal propensity (Southwood 1962, 1988). High levels of dispersal are favoured in ephemeral habitats, as an essential strategy for survival (Duputié and Massol 2013; Friedenberg 2003; McPeek and Holt 1992; Southwood 1962; Travis and Dytham 1999), but not in persistent habitats that do not exert such a selective pressure. Between these two extremes of the habitat durational axis, there is a wide spectrum of habitats with varying degrees of temporal persistence that exert different selective forces depending on the spatial configuration of the suitable habitat patches as well as on the requirements of the focal organism in space and time (Southwood 1962, 1977). By comparing taxonomically related lineages of similar requirements associated with habitats that differ consistently in their relative durational stability and spatial continuity, Ribera and co‐authors (Ribera 2008; Ribera, Barraclough, and Vogler 2001; Ribera and Vogler 2000) proposed and evaluated a range of predictions about the effect of habitat association on the spatial distribution, population structure and speciation rates of habitat specialist lineages. The so‐called ‘Habitat Constraint hypothesis’ (‘HC hypothesis’ hereafter; alternatively cited by other authors as ‘*habitat persistence*’ or ‘*habitat stability*’ hypothesis) predicts that in dynamic habitat patches with relatively lower durational stability, the persistence of specialist lineages requires an increased frequency of inter‐patch migration events. The greater tendency for migration in such regimes will lead to wider and more dynamic geographic ranges and higher levels of gene flow limiting population subdivision. Vice versa, under regimes of greater habitat stability, lineage persistence does not require such dispersal dynamics. Stable habitat specialist lineages may thus exhibit limited dispersal, which is evident from smaller species ranges, reduced gene flow among populations and higher speciation rates as a result of increased geographic isolation (Ribera, Barraclough, and Vogler 2001; Ribera and Vogler 2000).

Several empirical studies have observed habitat‐induced differences in phylogeographic structure or inter‐population genetic divergence between closely related lineages associated with distinct habitat types, which were consistent with the HC hypothesis (e.g., water beetles: Abellán, Millán, and Ribera 2009; Hjalmarsson, Bergsten, and Monaghan 2015; Lam, Gueuning, et al. 2018; Lam, Toussaint, et al. 2018; terrestrial beetles: Caterino, Chatzimanolis, and Richmond 2014; Papadopoulou et al. 2009; mayflies: Drotz et al. 2012; birds: Harvey et al. 2017; Johnson et al. 2023; multi‐taxon: Marten, Brändle, and Brandl 2006; and flatworms: Rader, Unmack, and Moore 2017). Nevertheless, a few authors have questioned the validity of the HC hypothesis, as they have found sympatric stable habitat taxa to present widely variable genetic metrics and population structuring (see Murphy, Guzik, and Wilmer 2010; Short and Caterino 2009). Such variation, however, might be attributed to the proposed asymmetry of habitat constraints (see Ribera 2008), that is, the assumption that in stable habitats, additional ecological or geographic factors may affect connectivity patterns resulting in a range of outcomes, in contrast to dynamic habitats where inter‐patch movement is necessary for lineage survival and thus higher predictability is expected. Appropriate empirical systems are needed to establish the extent of this potential asymmetry, beyond the lotic versus lentic paradigm in freshwater systems that has been the focus of the majority of the existing studies.

Most of the abovementioned studies that evaluated the evolutionary predictions of the HC hypothesis, with few exceptions, focused on dispersive (mostly winged) lineages, where there was an identifiable trait (e.g., wing loading) that favoured dispersal in the dynamic habitat type. It is still unclear if similar patterns should be expected in dispersal‐limited taxa that do not present such traits and might thus be more prone to local extinction. Frequent local population extinction and recolonisation of dynamic habitat patches may have important demographic and genetic consequences (McCauley 1991; Reigada et al. 2015) and thus could also affect the evolutionary trajectories of habitat‐specialist lineages in a predictable way. A growing body of literature is now highlighting the previously neglected importance of population extirpation, alongside that of population isolation, in controlling speciation rates (Dynesius and Jansson 2013; Harvey, Singhal, and Rabosky 2019; Li et al. 2018), which could provide an alternative explanation for lower speciation rates in dynamic habitat lineages. We here focus on a pair of closely related insular lineages of flightless beetles, which are associated with distinct habitat types and differ consistently in their phylogeographic patterns (Papadopoulou et al. 2008), although they are uniform morphologically, that is, they do not show any obvious differences in their morphological traits that could be linked to dispersal ability.

**FIGURE 1:**
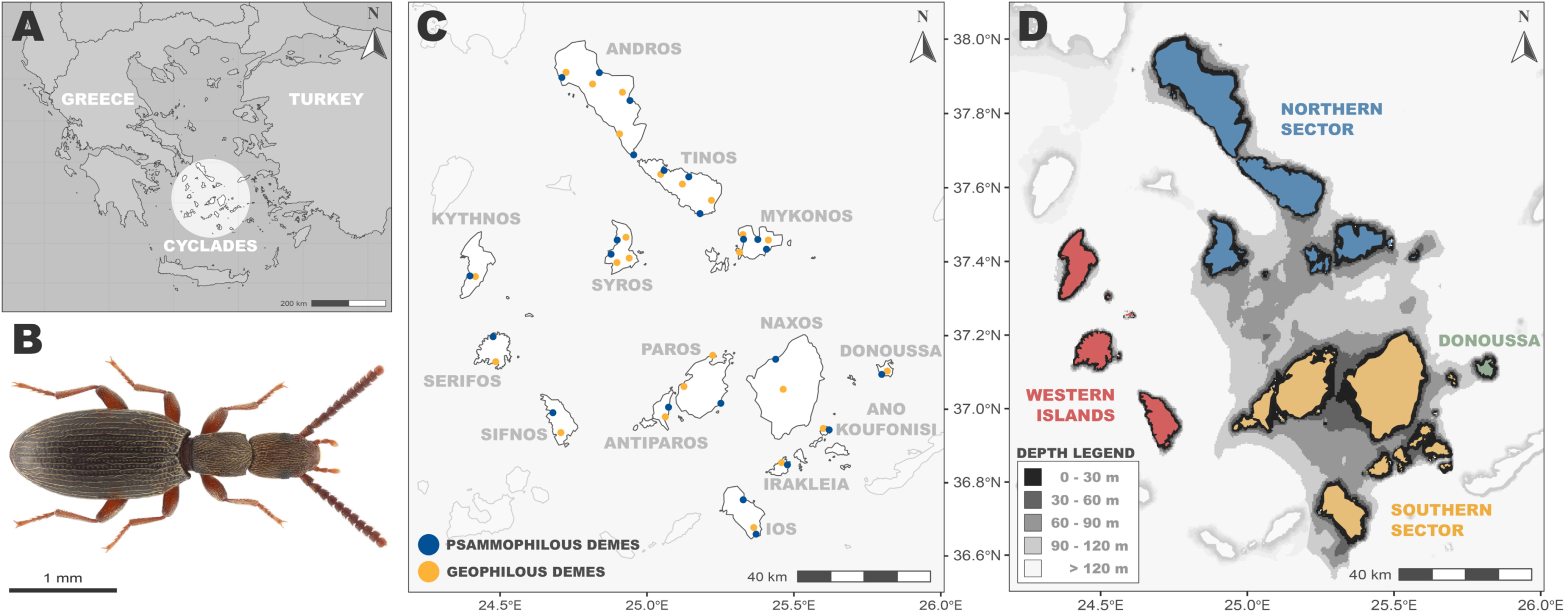
(A) Location of the Cyclades insular system within the Aegean archipelago in the Eastern Mediterranean. (B) Image of *Eutagenia smyrnesis* (Solier 1838) specimen (Photo credit: Christodoulos Makris). (C) Sampling scheme across 14 islands in the Cyclades. Blue dots represent the dynamic coastal sand dune habitat type, while yellow dots correspond to the stable compact soil habitat localities. (D) Bathymetry map of the focal area. Different shades of grey represent the assumed topography of the area during the low seal‐level periods, based on contemporary bathymetry. Geospatial data were retrieved from the Eurostat—GISCO (the Geographic Information System of the Commission) database using the *giscoR* package (Hernangómez 2023). Bathymetric data were downloaded from the NOAA (National Oceanic and Atmospheric Administration) database, as implemented in *marmap* R package (Pante and Simon‐Bouhet 2013).

The focal *Eutagenia* spp. (Coleoptera: Tenebrionidae: Pimeliinae) are small‐sized (2.5–3.5 mm), flightless, soil‐dwelling beetles with hypogeal larvae, widely distributed across the Cyclades archipelago in the Aegean Sea (Greece; see Figure 1A). They occupy uniform trophic and climatic niches (detritivores, adapted to arid/semiarid climatic conditions) and have equally limited dispersal capabilities (lack of hind wings and fused elytra, indistinguishable body size and shape). As their life cycle is tightly linked to the soil, edaphic factors may be major determinants of their habitat preference, spatial distribution and evolutionary history (e.g., see Cheli et al. 2021; De Los Santos et al. 2000). Indeed, while all populations across the Cyclades were initially considered as belonging to a single species, *Eutagenia smyrnensis* (Solier, 1838), due to their largely uniform external morphology (see Figure 1B), molecular analyses recovered two highly divergent clades with distinct soil substrate preference (and consequently habitat association) and contrasting phylogeographic patterns. The sand‐obligate ‘psammophilous’ clade is distributed in sandy beaches and coastal sand dunes and presents little phylogeographic structure and very shallow mtDNA genealogies across the archipelago, indicating some form of inter‐island dispersal. On the contrary, the compact soil (clay or silt) ‘geophilous’ clade is distributed in phrygana (garrigue) shrublands and semiarid grasslands and is subdivided into several divergent lineages, each geographically confined to a small group of adjacent islands (Papadopoulou et al. 2008, 2009; see Figures S11 and S12 in Appendix S1). The striking phylogeographic differences between the two habitat‐specialist clades of *Eutagenia* spp. were further mirrored in other strictly psammophilous and geophilous flightless soil‐dwelling darkling beetle genera across the Cyclades, which was attributed to presumed differences in the durational stability of the two habitat types (Papadopoulou et al. 2009).

Sandy shores are geologically young and dynamic systems, which are continuously reshaped by aeolian and marine processes that drive an equilibrium between erosion and sediment deposition (Farrell et al. 2023; Sherman 1995; Sherman and Bauer 1993). Despite some variation in the durational stability of sandy habitat patches depending on the landform type (decades to thousands of years; Davidson‐Arnott 2010), in a geological timescale they are considered short‐lived systems that can grow, reform, recover or disappear in response to both stochastic (e.g., storms; Davidson‐Arnott 2010; Defeo et al. 2009; Mathew, Davidson‐Arnott, and Ollerhead 2010; McLachlan and Defeo 2018) and cyclical events (e.g., Quaternary climatic and sea‐level changes). During the glacial–interglacial cycles, marine and aeolian processes redistribute the sediments and drive cycles of destruction and reconstruction of the sandy habitat patches along the coastline (Arce‐Chamorro et al. 2023; Maun 2009; Ritchie 1972). This cyclical process and thus the fairly short‐term configuration of coastal sand dunes has been considered as an important driver of the low fauna endemicity observed in these systems (McLachlan 1991). In the Cyclades archipelago, intense changes in coastline topography and island connectivity have been inferred during glacial–interglacial periods (e.g., Lykousis 2009; Simaiakis et al. 2017), which may have further amplified this restructuring process, as well as its potential effects on the psammophilous biota. The dynamic nature of the sand dune habitats contrasts with the relatively more stable compact soil habitats (predominantly phrygana shrublands and semiarid grasslands), which are distributed widely across the entire island surface and are thus expected to exhibit higher spatial and temporal continuity over longer timescales across the Cyclades islands. Despite cycles of expansion and contraction in island area during the Quaternary and possible changes in flora composition and/or distribution, it can be assumed that the compact soil substrate remained continuously available to geophilous lineages for millions of years as the Cyclades have not been totally submerged since their original formation in the Late Oligocene/Early Miocene (~23 Ma; reviewed in Fassoulas 2018).

The combination of the aforementioned organismal and habitat attributes with the inherent properties of an archipelago provides an ideal system to assess the original predictions of the HC hypothesis on dispersal‐limited taxa while taking into account the asymmetry of habitat constraints and the potential effects of extinction–recolonisation dynamics. First, for flightless terrestrial organisms, multi‐island systems provide spatial mosaics of easily defined habitable areas (i.e., insular landmass) divided by an inhospitable matrix (i.e., surrounding water) (Warren et al. 2015). Across the Cyclades islands, the presumably strong inter‐island gene flow barriers are common for both habitat types, thus allowing the evaluation of the cornerstone assumption that dispersal is favoured in geologically dynamic habitats. Second, the focal islands are considerably homogeneous in terms of climatic (i.e., thermo‐Mediterranean bioclimatic zone; Yassoglou, Tsadilas, and Kosmas 2017; low annual precipitation of ~400 mm/year; Giannikopoulou 2014) and topographic (i.e., relatively small islands without extreme altitudes) conditions. This allows treating the islands as natural (pseudo)replicates for evaluating the consistency and uniformity of the observed patterns across the archipelago. Third, the Cyclades have been significantly affected by Quaternary sea‐level fluctuations (Simaiakis et al. 2017), which led to repeated isolation–reconnection cycles among some (but not all) of the focal islands. Specifically, most of the western islands remained completely isolated from each other and detached from neighbouring land masses since the penultimate glacial period (~140 Ka BP), while the rest of the Cyclades Plateau formed a large ‘palaeo‐island’ during the Last Glacial Maximum (LGM; 30–18 Ka BP) that was progressively fragmented following the sea‐level rise. Due to the complex seabed topography, there was great variation in the timing and spatial configuration of inter‐island land bridges (see Figure 1D for island groups based on palaeogeography/bathymetry; Kapsimalis 2009; Lykousis 2009), which provides the opportunity to assess the stringency of habitat constraints across island pairs with varying degrees of connectivity. Such a dynamic geological history is expected to have a major impact on the evolutionary dynamics of insular lineages (e.g., Freitas, Campos, and Araujo 2024; Papadopoulou and Knowles 2015, 2017), but the combined effects of such processes with habitat constraints remain unexplored.

We here use genome‐wide SNP data to assess the predictions of the HC hypothesis across the dynamic archipelago of the Cyclades at the population genomic level in flightless darkling beetles of the genus *Eutagenia*. By conducting comparative population genomic and demographic analyses between psammophilous and geophilous demes that are co‐distributed across the Cyclades, we aim to evaluate the microevolutionary predictions of the HC hypothesis. First, we test the core expectation that lineages associated with dynamic habitats show higher population connectivity. If the hypothesis stands, we expect consistently increased inter‐island gene flow between psammophilous demes in comparison to their geophilous counterparts, despite being constrained by the same dispersal barriers. Second, we assess the alternative—but not mutually exclusive—hypothesis that deme persistence in dynamic habitats is affected by relatively frequent extinction and recolonisation events. In this scenario, we expect strong bottleneck events to be prevalent in the demographic history of psammophilous demes, but mostly absent for the geophilous clade. Third, we consider the proposed asymmetry in habitat constraints between the two contrasting types. We expect that the geophilous demes should present more variable and island‐specific demographic patterns, reflecting the historical configuration of the Cyclades. Conversely, the dynamic habitats exert pressure on local populations universally and thus should lead to more homogeneous demographic histories that may not adhere to palaeogeographical expectations.

## 2 Methods

### 2.1 Sampling and Library Preparation

Sampling was conducted across 14 Cyclades islands (Table S10) using baited pitfall traps and hand collection. The sampling scheme aimed to (a) include islands of different palaeogeographical history and degree of isolation, considering the Quaternary isolation–reconnection cycles, (b) create a correspondent grid of geophilous and psammophilous demes across all islands (i.e., the number of demes and their pairwise Euclidean distances to be as similar as possible for the two clades) and (c) sample multiple demes from selected islands to allow for inter‐ versus intra‐island comparisons. In total, 24 geophilous and 23 psammophilous demes were successfully sampled (Figure 1C). Specimens were preserved in 100% ethanol and stored at −20°C. DNA extraction was performed following a commercial bead‐based protocol (Biosprint 96 DNA Blood kit, Qiagen) as implemented in the automated KingFisher Flex System (Thermo Fisher Scientific).

Library preparation followed the double‐digest restriction site–associated DNA sequencing (ddRADseq) protocol of Peterson et al. (2012) with minor modifications (see Papadopoulou and Knowles 2017), for a total of 339 specimens. Briefly, DNA was double‐digested with restriction enzymes EcoRI and Msel, unique barcodes (10 bp) and adaptors were ligated to the digested fragments and the individually barcoded samples were pooled into libraries of 72–80 samples each. Each library was size selected for fragments of 350–450 bp using a Pippin Prep Instrument (Sage Science Inc.). Following size selection, the fragments were PCR amplified (8–10 amplification cycles) using high‐fidelity DNA polymerase (iProof, Bio‐Rad) and sequenced on the Illumina HiSeq2500 platform (single‐end, 150 bp reads) at the Centre for Applied Genomics (SickKids, Toronto, Ontario, Canada).

### 2.2 Data Processing and Genotype Calling

*De novo* assembly of the raw Illumina reads to putative loci and genotype calling was performed using the STACKS2 v2.64 pipeline (Rochette, Rivera‐Colón, and Catchen 2019). Initially, the *process_radtags* program was used for demultiplexing and quality filtering. Approximately 20% of raw read per library were discarded due to low quality (Phred score < 10, using a sliding window of 15%), detection of ambiguities, failure to pass the Illumina chastity filter or a mismatch of the barcode or adapter by more than 1 bp. Following the quality filtering step, six individuals that retained less than 200,000 reads or less than 80% of their initial amount of reads were discarded. The retained reads were examined using FASTQC (Andrews 2010) and MULTIQC (Ewels et al. 2016) and trimmed (5 bp at the 5′‐end and 24 bp at the 3′‐end) using SEQTK (https://github.com/lh3/seqtk). The processed reads were divided into two batches based on habitat type. We chose to proceed with different core assembly parameters for each batch (i.e., the *M* and *n* parameters; indicating distance within and between loci, respectively) due to pronounced differences in trial runs of the *r80 method* of parameter optimisation (Paris, Stevens, and Catchen 2017; Rivera‐Colón and Catchen 2022; Rochette and Catchen 2017). The *r80 method* identified the *M* = 5 and *n* = 6 combination to be optimal for the geophilous dataset, and the *M* = 3 and *n* = 3 combination for the psammophilous dataset. The remaining parameters of the pipeline were kept as default with few exceptions, as follows: (a) we set the minimum depth of coverage parameter (*m*) to a value of 5, (b) we decreased the genotyping model alpha value (from 0.05 to 0.01) to increase confidence in genotype calling, and (c) we decreased the high‐coverage threshold (3.0 to 2.0 SD units) and (d) activated the deleverage algorithm, to mitigate the formation of overmerged clusters. Following optimisation, 15 problematic samples were identified and discarded based on mean sequencing depth and relative proportion of missing data (see Cerca et al. 2021), as estimated using VCFTOOLS v0.1.16 (Danecek et al. 2011). Minimally filtered datasets of at least 30% locus coverage over all individuals within each habitat batch were generated using the *populations* program.

Three additional filtering steps were performed on the exported datasets to remove loci that were potentially (a) under selection or (b) overmerged, as well as to mask (c) variants with low or artificially high depth of coverage. Specifically, loci that were potentially under selection were detected using BAYESCAN v2.0 (Foll and Gaggiotti 2008). The minimally filtered VCFs were thinned to one variant per RAD locus and converted to Bayescan input files using the PGDSPIDER v2.1.1.5 (Lischer and Excoffier 2012). We kept the default settings for the chain attributes and the model, except for the prior odds parameter (*PO*) which was set to a value of 1000. Outliers were identified within the R programming environment (R Core Team 2022) using the *plot_bayescan* function of the *plot_R.r* script with an FDR threshold of 0.05, as provided by the authors (https://github.com/mfoll/BayeScan/tree/master/R_functions). Convergence was evaluated by both visual inspection of the trace plots and diagnostic tests applied using the *coda* R package (Plummer, Best, and Cowles 2006). Overall, a total of 98 loci from the geophilous dataset and 415 for the psammophilous dataset were identified for removal. Subsequently, potentially overmerged loci were detected using site variability per locus as a proxy. We used a custom script to calculate a locus‐specific theta value based on the number of segregating sites. We considered a locus as an outlier if its theta value was higher than the 99.5 percentile of the estimated theta distribution (see Figure S1). This threshold was considered the most appropriate to be applied for both habitat batches regardless of their differences in theta distribution. This step identified 82 loci in the geophilous dataset and 101 in the psammophilous dataset as potentially overmerged clusters. For each habitat batch, the two outlier lists (loci potentially under selection and overmerged clusters) were combined into a blacklist file and excluded in a second run of the *populations* program within the STACKS2 pipeline. The output VCF files included both variant and invariant sites (*‐‐vcf‐all* option in *populations* program). Finally, we masked specific variants by converting them to missing data based on their mean depth of coverage (DP). We used the *vcfR* package (Knaus and Grünwald 2017) to exclude lower‐quality variants (i.e., variants with < 5X coverage) and artificially high‐DP variants (i.e., variants with mean DP value higher than the 97.5 percentile of the sample‐specific DP distribution, thus likely to originate from overmerged clusters), which were not detected in prior filtering steps.

The two filtered datasets (one per habitat type) were further processed for downstream analyses. Final filtering on missing data, minor allele frequency and thinning to a single variant per RAD locus were applied using the *SNPfiltR* R package (DeRaad 2022), depending on the requirements of each analysis (see details within each of the following sections). For thinning, we chose to keep the first variant per locus instead of a random one for reproducibility purposes. All file conversions were done using the PGDSPIDER software, unless stated otherwise.

### 2.3 Genetic Diversity, Differentiation and Divergence

Number of private and polymorphic sites per deme were calculated in STACKS2. Genetic diversity (*π*) per deme, as well as pairwise genetic differentiation (*F*_ST_) and genetic divergence (*D*_XY_) among demes, were estimated using both variant and invariant sites, as implemented in the PIXY v.1.2.7 software (Korunes and Samuk 2021; Samuk 2023). To calculate genetic diversity, we considered variant and invariant sites (i.e., ‘genome‐wide’ or ‘autosomal’ genetic diversity; see Schmidt et al. 2021) that were present in at least 50% of individuals within each deme. Each deme was filtered independently to avoid any bias in the calculation of genetic diversity values due to uneven exclusion of loci in cases of high divergence (see Figure S2). Genetic divergence and differentiation indices were calculated using loci with sites that were shared across 70% of the individuals within each habitat type. Note that for all metrics, demes with less than five individuals were excluded from the final comparisons.

### 2.4 Genetic Structure

Genetic clustering of the sampled individuals was inferred using unlinked variants (i.e., a single variant per RAD locus), after excluding singletons (i.e., minor allele count < 3; see Linck and Battey 2019) and variants that were not present in at least 80% of individuals. We applied a model‐based clustering method as implemented in the Bayesian algorithm STRUCTURE v.2.3.4 (Pritchard, Stephens, and Donnelly 2000). We run STRUCTURE separately for each habitat type for *K*‐values ranging from 1 to 10, with 20 independent replicates per *K*. Each independent replicate was run for 200,000 MCMC simulations with a burn‐in of 100,000 iterations, without using any sample group information to assist the inference of clusters. For each *K*‐value, we retained the 10 replicates with the highest estimated likelihood. The most appropriate *K* to describe our data was chosen based primarily on the Δ*K* method (Evanno, Regnaut, and Goudet 2005); however, as Δ*K* cannot identify panmixia (i.e., *K* = 1), we compared the results with those from the lnP(*K*) estimate, as recommended (e.g., Gilbert 2016). The calculation of statistics across the different *K*‐values and the visualisation of the barplots were performed using STRUCTURE HARVESTER (Earl and vonHoldt 2012) and STRUCTURE SELECTOR (Li and Liu 2018). The results were evaluated by comparing with PCA analyses of the same datasets (see recommendation by Linck and Battey 2019), as implemented in the *adegenet* R package (Jombart 2008).

### 2.5 Genealogical Inference

Evolutionary relationships among demes within each habitat type were inferred under the coalescent model, using the SVDQuartets method (Chifman and Kubatko 2015) implemented in PAUP* v.4 (Swofford 2003). For each habitat dataset, we included one deme of the opposite habitat type as an outgroup. Variants that were not present in at least 30% of individuals and singletons were excluded, and the datasets were thinned to one variant per RAD locus. To prepare the input files for SVDQuartets, the VCF files were converted to NEXUS format using the *vcf2phylip* v.2 python script (Ortiz 2019) with default options. All possible quartets were evaluated and clade support values were estimated with 500 nonparametric bootstrap replicates.

### 2.6 Demographic Modelling

Local population isolation and local population persistence were evaluated using demographic analyses based on the site frequency spectrum (SFS) as implemented in FASTSIMCOAL2 v.27093 (Excoffier et al. 2013, 2021). Population isolation was evaluated by modelling differences in the existence and persistence of gene flow through time. We constructed 4 two‐deme models that favoured either isolation (complete or contemporary isolation models) or contact between demes (contemporary or continuous migration models), and tested them on both intra‐ and inter‐island combinations, covering a wide range of barrier intensity against dispersal. All models simulated demes with constant post‐divergence effective population size and only differed in the presence and duration of (symmetrical) gene flow. The isolation models described two demes that either diverged and evolved without any exchange of migrants (complete isolation) or underwent a period of post‐divergence gene flow that was interrupted at some point in time (contemporary isolation). To ensure that the two models remained distinct during parameter estimation, we set a relative lower and upper bound for the cessation time point of gene flow (*T*_INT_), forcing a minimum period of early contact that could take values up to half of the post‐divergence time (0.5 × *T*_DIV_ ≤ *T*_INT_ ≤ 0.9 × *T*_DIV_). The migration‐favouring models were also structured in a similar manner, with either uninterrupted post‐divergence gene flow (continuous migration) or a minimum period of contemporary contact that was allowed to fluctuate as far back as half of the post‐divergence time (0.1 × *T*_DIV_ ≤ *T*_INT_ ≤ 0.5 × *T*_DIV_; contemporary migration). A visual representation of the models can be found in Figure 4. Note that migration rate (proportion of migrants per generation) is also constrained to a minimum value of 5 × 10^−7^ to ensure a significant amount of gene flow when including it as a model parameter (the specific value was chosen based on the simulations of Papadopoulou et al. 2008). To explore differences in population persistence, we considered fluctuations in effective population size (*N*_e_) as a proxy. We constructed a set of four single‐deme models that represented either high (constant *N*_e_ or deme expansion) or low persistence (deme contraction or bottleneck; see visual representation in Figure 5). All models included a common resize parameter which controlled an instantaneous *N*_e_ change. For the constant *N*_e_ model, we allowed for a minor fluctuation (10% increase or decrease), while we constrained the rest of the models to only consider strong changes in *N*_e_ of at least two orders of magnitude.

For both types of demographic analyses (single‐ or two‐deme), we only included localities with at least seven sampled individuals, confining the analyses to 35 demes (17 geophilous and 18 psammophilous). For the two‐deme models, we considered all intra‐island pairings within each habitat that met the above requirement, while we selected a subset from the available inter‐island combinations after keeping a maximum of 2 demes per island. We estimated the folded SFS (or folded joint SFS for two‐deme analyses) using the *easySFS* python script (https://github.com/isaacovercast/easySFS), as there was no available information on the derived and ancestral allelic state. For each SFS/jSFS calculation, we only used unlinked variants that were present in at least 70% of individuals included in the respective deme/deme pair. To mitigate the effect of any remaining missing data, each SFS was projected down (see the DADI manual; Gutenkunst et al. 2009) to 5–7 individuals and each jSFS to 6 individuals per deme. Furthermore, deme pairs with less than 2000 variants per deme were discarded from further analyses, totalling a final number of 101 intra‐ and inter‐island deme pairs (59 psammophilous and 42 geophilous pairs).

Model parameters were estimated in FASTSIMCOAL2 without including invariant sites. Hence, our parameter estimates are scaled based on a fixed value of contemporary *N*_e_ of either the focal deme in the single‐deme models or one of the two demes in the two‐deme models. The fixed effective population size (*N*_e_) was estimated based on the *N*_e_ = (*θ/4 μ*) equation, approximating *θ* using the nucleotide diversity (*π*) estimates as calculated in PIXY (see Section 2.3) and an assumed mutation rate per site per generation (*μ*) of 2.8 × 10^−9^ (Keightley et al. 2014, 2015). To estimate the model parameters and maximise the composite likelihood, each model for each dataset was run with 100 replicates, considering 500,000 simulations and 100 expectation conditional maximisation (ECM) loops per replicate. The best model to describe the data was determined within an information theoretic framework (Burnham and Anderson 2002; e.g., Thomé and Carstens 2016), using the Akaike information criterion (AIC). Briefly, for each case and each tested model, the replicate that maximised the likelihood was selected and used to calculate its AIC score. For the models under comparison, the scores were rescaled by calculating their difference (ΔAIC) against the best model (i.e., the one with minimum AIC value), and subsequently their relative likelihood and AIC weight. We did not estimate confidence intervals through a bootstrapping procedure or transform any of the inferred parameters into biologically meaningful estimates, as we did not have accurate clade‐specific mutation rates and thus the estimated values could be misleading. Regardless, our primary objective was to identify and compare the predominant demographic events that shaped the observed allele frequency spectra within populations from the two habitat types, rather than estimating the precise demographic parameters of each deme.

## 3 Results

### 3.1 Illumina Data Processing

A total of 318 samples with an average of 1,600,000 reads per sample (min = 471,046; max = 5,897,025) and mean depth values that ranged between 18 and 77 were retained for the remaining filtering steps and subsequent analyses (Table S1). Specifically, the psammophilous dataset included 166 individuals from 23 demes in 14 islands, with a total of 19,597 polymorphic loci (50,543 biallelic variants; 35% missing data). Each psammophilous deme had an average of 2,753,776 sites (SD = 111,971). Similarly, the geophilous dataset included 152 individuals, sampled from 24 localities/demes of the same 14 islands, with 19,294 polymorphic loci (261,060 biallelic variants; 46% missing data). The majority of geophilous demes retained less (but comparable) sites than their psammophilous counterparts (*M* = 1,914,724; SD = 288,889). However, we observed a downward bias in the western island geophilous demes (*M* = 721,700; SD = 29,465), even though our sequencing (i.e., multiple libraries) and quality control schemes did not point to technical problems. Indeed, when we analysed the western demes separately and repeated the assembly and filtering steps, we recovered an amount of total sites per western deme (*M* = 2,068,413; SD = 60,999) that better resembled the rest of the geophilous dataset (*M* = 2,386,579; SD = 148,751). As allelic dropout increases with evolutionary distance (Huang and Knowles 2016; Leaché and Oaks 2017), the aforementioned pattern is the result of significant divergence between western and eastern (i.e., Northern and Southern sector islands, and Donoussa; see Figure 1D) demes in the geophilous dataset. We did not observe any analogous pattern in the psammophilous data.

### 3.2 Metrics of Local Genetic Diversity, Differentiation and Divergence

**FIGURE 2:**
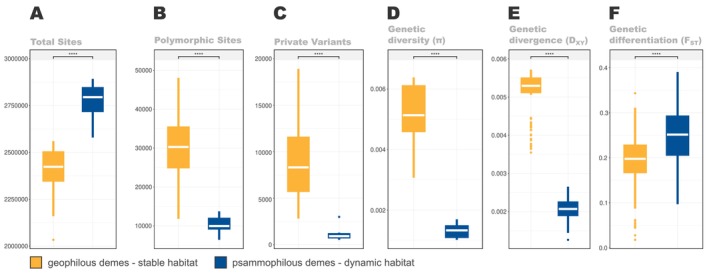
Comparison of the distribution of different assembly metrics and population genetic statistics between stable (geophilous) and dynamic (psammophilous) habitat demes. (A–C) Boxplots representing the distribution of different assembly metrics as estimated in STACKS2. For all metrics, deme‐specific values were calculated after averaging across all individuals within each deme. (D–F) Boxplots representing the distribution of average genetic diversity, divergence and differentiation values, respectively, as estimated in PIXY. Deme‐specific values were calculated after averaging across all loci (i.e., PIXY ‘windows’) within each deme, following the recommended approach (see https://pixy.readthedocs.io/en/latest/index.html). Statistical significance (**** for *p* < 0.0001) on top of each plot was estimated using Welch's *t*‐test as implemented in the *rstatix* R package.

In accordance with our observations during the assembly process, combined analyses that included all geophilous demes, identified disproportionately high values of genetic divergence (*D*_XY_) and differentiation (*F*_ST_). Specifically, both inter‐island *D*_XY_ and *F*_ST_ pairwise values were on average three times higher in those comparisons involving demes sampled from eastern and western islands (see Figures S3 and S4, and Tables S4 and S5). We thus, conservatively chose to only consider genetic estimates measured for the eastern geophilous demes when comparing the two habitat types (i.e., geophilous vs. psammophilous comparisons in Table 1 and Figure 2), to avoid biases resulting from the inclusion of the western geophilous demes that may comprise a highly diverged incipient species or even a distinct taxon.

**TABLE 1:** Mean values and standard deviation of assembly metrics and population genetic statistics, as estimated for the geophilous and psammophilous demes. Comparison of mean values was performed using Welch's *t*‐test as implemented in the *rstatix* v.0.7.2 (Kassambara 2023) R package.

Parameter	Habitat type				Welch's *t*‐test	
	Geophilous		Psammophilous			
	Mean	SD	Mean	SD	*t*	*p*
Total sites	2,386,579	148,751	2,774,346	93,110	−9.15	3.02 × 10^−9^
Polymorphic sites	31,491	9207	10,430	1883	9.01	1.18 × 10^−7^
Private sites	9238	4591	1025	498	7.12	3.15 × 10^−6^
*π*	0.0052	0.0009	0.0013	0.0002	16.05	2.76 × 10^−11^
*D*_XY_	0.0051	0.0005	0.0021	0.0003	57.24	6.33 × 10^−104^
*F*_ST_	0.19	0.06	0.25	0.06	−8.01	6.02 × 10^−14^

Pairwise genetic divergence was consistently higher for the geophilous demes when compared to their psammophilous counterparts. Specifically, *D*_XY_ estimates for the geophilous deme pairs were on average twice as high as those measured for the psammophilous pairs. However, the genetic differentiation (*F*_ST_) of psammophilous deme pairs was slightly but significantly higher than that of the included geophilous demes.

Estimates of genetic diversity were also consistently different among the two habitat types (deme‐specific values in Tables S2 and S3). On average, we identified three times more polymorphic sites and nine times more private sites per geophilous deme in comparison to their psammophilous counterparts. Similarly, mean genetic diversity (*π*) per deme was on average five times higher in the geophilous lineage. Interestingly, the variation among demes for the aforementioned estimates was also higher in the geophilous lineage.

### 3.3 Evaluation of Genetic Structure

**FIGURE 3:**
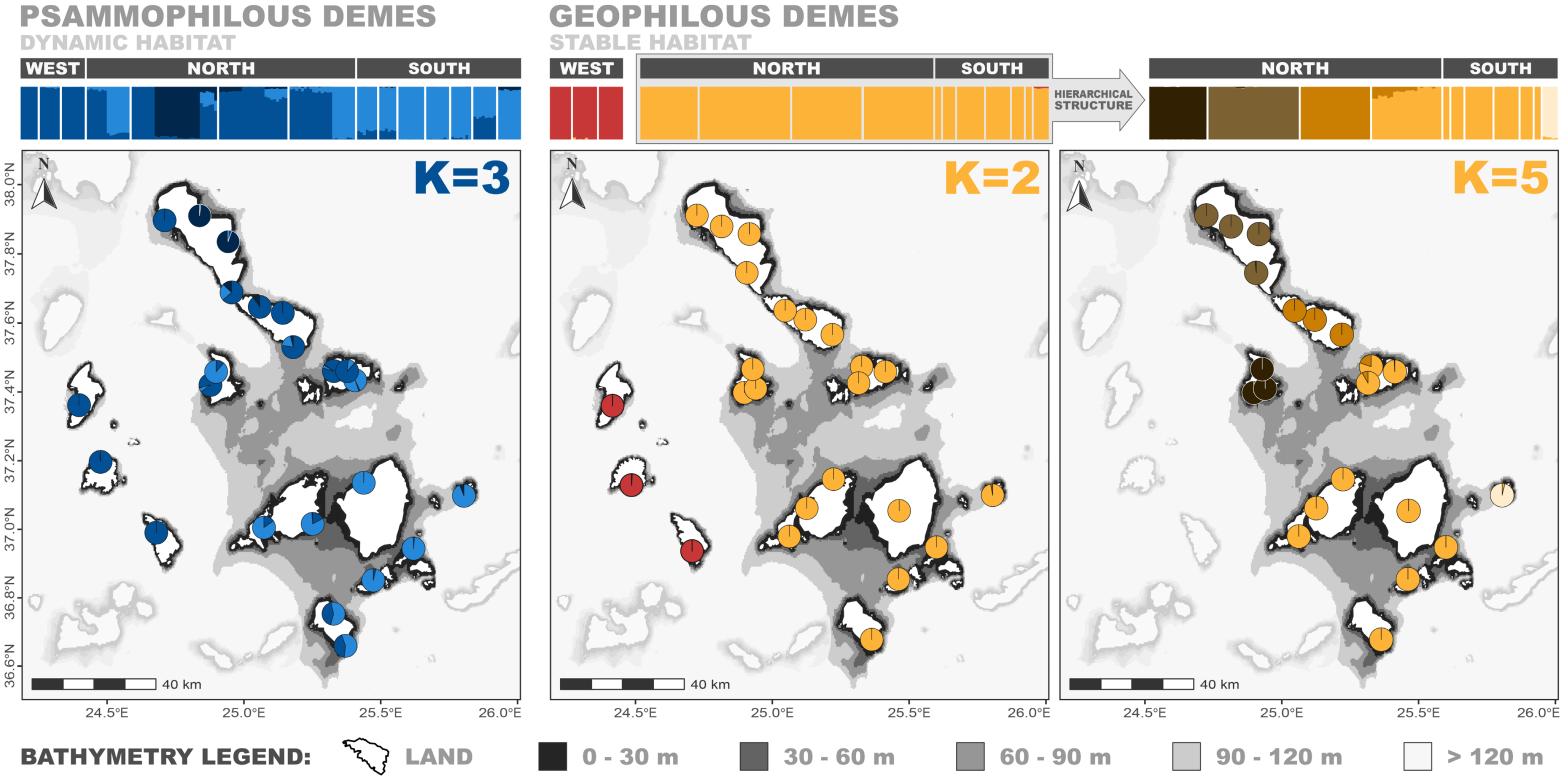
Spatial distribution of genetic clusters as inferred by the STRUCTURE Bayesian algorithm. Each individual in the barplots is represented by a vertical bar, while different islands are delimited by thin white stripes. For each habitat type and barplot, a bathymetry map is provided, depicting average membership probabilities of local populations as pie charts.

STRUCTURE analyses detected contrasting patterns between the two habitat types. For the geophilous dataset and based on 2192 unlinked variants (11% missing data), Δ*K* peaked at *K* = 2, while LnP(*K*) increased steadily until *K* reached values of 5–6 (Figure S5). Inspection of the STRUCTURE plots across the different *K* values revealed that the continuous increase in LnP(*K*) was due to substructure within the genetic cluster that included the eastern demes (Figure S7). Hence, we split the dataset and reran STRUCTURE including only these demes (3247 unlinked variants; 10% missing data). This second run identified that Δ*K* peaked at *K* = 5 and LnP(*K*) reached a plateau at the same *K* value, delineating 5 genetic clusters that corresponded well to both current and historical geography of the region. The majority of northern islands and the island of Donoussa split into island‐specific clusters, while the southern sector islands grouped into a single uniform cluster. For the northern sector, one exception was the island of Mykonos which was primarily grouped with the southern islands (Figures 3 and S8). The choice of *K* value was not as straightforward for the psammophilous dataset. Based on 5005 unlinked variants (8% missing data), Δ*K* peaked at *K* = 3, while LnP(*K*) did not reach a plateau at any *K*‐value (Figure S5). Inspection of the inferred plots across *K* values did not show any sign of (geographic) substructure, while the complexity of membership probabilities per local population increased alongside the increase in *K* (Figure S6). If plotted for *K* = 3, the spatial distribution of genetic clusters was only moderately divided geographically (Figure 3).

### 3.4 Evolutionary Relationships of Local Populations

The evolutionary relationships among geophilous and psammophilous demes were inferred in SVDQuartets using 16,876 and 14,292 unlinked variants, respectively. For the geophilous dataset, the estimated topology (Figure S10) was in accordance with both the palaeogeography of the archipelago and the clustering analyses. At the base of the tree, the isolated western islands formed an early diverged clade, while the majority of the remaining localities were grouped in strongly supported island‐specific clades. An exception was observed for the southern sector, which formed a single multi‐island clade with unresolved inter‐island relationships, reflecting their shared history as a single entity (up to ~8–13 Ka BP) and high levels of gene flow. Regarding the relationships between the main inferred clades, few cases of weak support (< 95% bootstrap) were observed.

For the psammophilous dataset, there was no apparent geographic structure across the tree, as even localities within the same island did not group together (Figure S10). Support values were low throughout the tree (with few exceptions), indicating the lack of phylogenetic structure and thus limited utility of depicting the relationships in the form of a bifurcating tree.

### 3.5 Inference of Demographic Patterns

#### 3.5.1 Population Isolation

**FIGURE 4:**
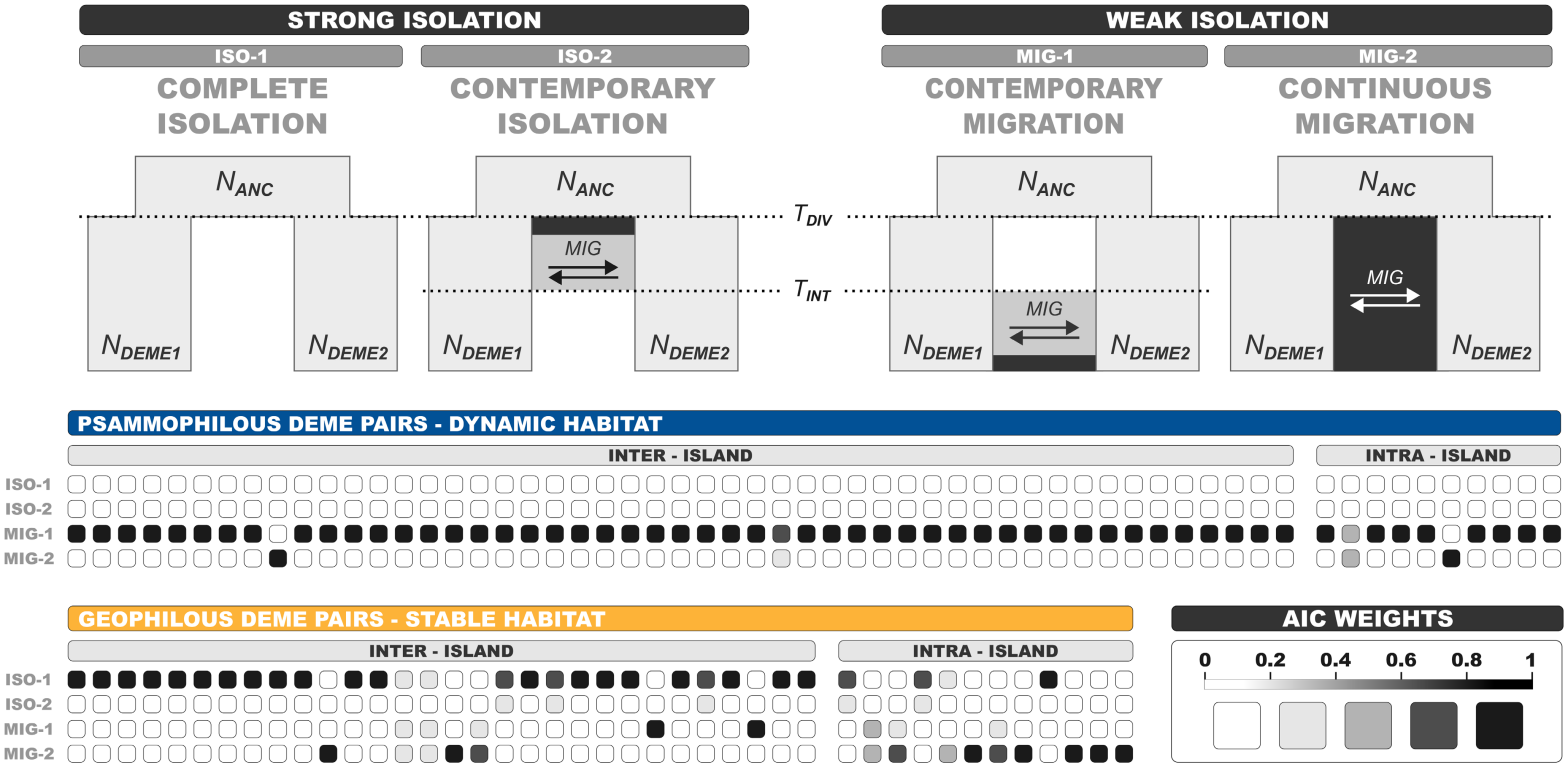
Results of two‐population demographic models assessing local population isolation. The top panel is a schematic representation of the four alternative two‐deme models. All models represent an ancestral population with effective population size *N*_ANC_ diverging at time *T*_DIV_ into two daughter demes with constant effective population sizes *N*_DEME1_ and *N*_DEME2_, respectively, without any loss or gain of individuals. Gene flow between demes is modelled as a symmetric exchange of migrants with rate MIG ≥ 5 × 10^−7^. When the time interval of contact is depicted as a black band, it symbolises a period during which migration is forced between the two demes, while a grey band depicts the limit that this interval is allowed to extend to. The interruption of gene flow is controlled by the parameter *T*_INT_, which is relative to the time of divergence (*T*_DIV_). For the contemporary isolation model (ISO‐2), 0.5 × *T*_DIV_ ≤ *T*_INT_ ≤ 0.9 × *T*_DIV_, while for the contemporary migration model (MIG‐1), 0.1 × *T*_DIV_ ≤ *T*_INT_ ≤ 0.5 × *T*_DIV_. The bottom panel presents the results of model selection as a heatmap, for the two habitat types. Each row in the heatmap represents a model, while each column corresponds to a two‐deme combination for which we simulated the four alternative demographic scenarios. The heatmap cells are coloured based on the AIC weight values on a scale from 0 (worst) to 1 (best) in intervals of 0.2 units, as depicted in the legend.

Demographic modelling of local population isolation inferred contrasting patterns of gene flow to be prevalent in the two habitat types. In the psammophilous datasets, the jSFS of all combinations was best explained by one of the two models that favoured contact between demes (i.e., contemporary and/or continuous migration). Specifically, the best model for the majority of combinations (95%) was that of contemporary gene flow, regardless of the pair origin (i.e., intra‐ or inter‐island pair) or the assumed intensity of dispersal barriers (Figure 4; Tables S8 and S9). On the contrary, multiple patterns were observed in the case of the geophilous demes. Within islands, the model selection step identified the contact models as the most probable for 75% of the pairs. For inter‐island pairs, gene flow patterns were driven by the intensity of geographic barriers. In the majority of population pairs (~75%), a model of complete isolation was selected, with few cases pointing to a brief period of historical gene flow (i.e., contemporary isolation model) as the second most likely model (Figure 4; Tables S8 and S9). The remaining inter‐island pairs that presented some kind of contact were either combinations of demes originating from the southern sector group (weak inter‐island isolation based on palaeogeography) or demes from neighbouring islands, in agreement with the inferred cluster membership probabilities from the STRUCTURE analyses.

#### 3.5.2 Population Persistence

**FIGURE 5:**
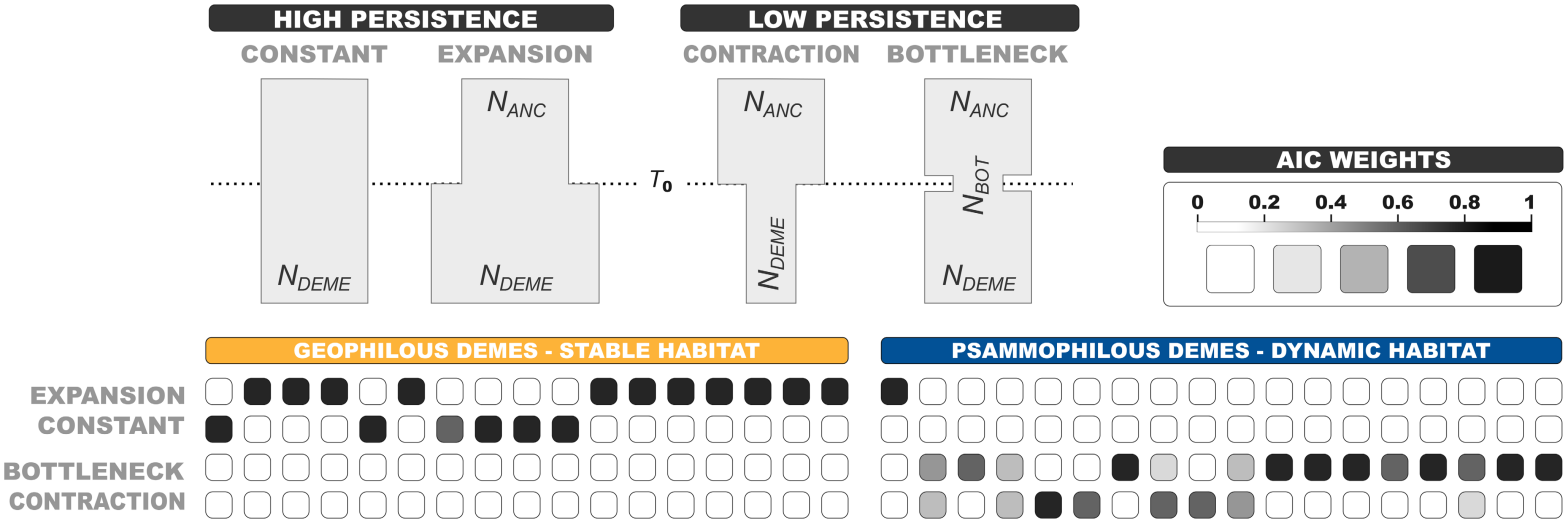
Results of single population demographic models assessing local population persistence. The top panel is a schematic representation of the four single‐deme models. All models represent a single deme through time that undergoes a single demographic event at time *T*_0_ ≤ 300,000 generations. The upper time limit of the demographic event was bounded to avoid simulating unreasonably high values that could potentially exceed the time of divergence of the deme of interest. All four models include a resize parameter controlling an instantaneous change of historical effective population size (*N*_ANC_), relative to a fixed contemporary size (*N*_DEME_). For the constant model, a resize event of nonsignificant change in size (0.9 × *N*_DEME_ ≤ *N*_ANC_ ≤ 1.1 × *N*_DEME_) was included. For the remaining three models, the change in size was considered significant only if it differed from the previous size by at least two orders of magnitude. Briefly and going backwards in time, the expansion scenario models a deme that at *T*_0_ resizes to *N*_ANC_ ≤ (10^−2^ × *N*_DEME_), while the opposite is true for the contraction scenario (i.e., *N*_ANC_ ≥ 10^2^ × *N*_DEME_). For the bottleneck scenario, *N*_ANC_ = *N*_DEME_, while *N*_BOT_ ≤ (10^−2^ × *N*_DEME_). The bottom panel presents the results of model selection for the two habitat types as a heatmap.

Demographic models that indicate higher local persistence were consistently selected for the stable habitat type. From a total of 17 geophilous demes, the expansion model was selected for 11 of them, while in the remaining 6, a constant *N*_e_ through time was inferred. On the contrary, of 18 psammophilous demes, 17 of them underwent either a bottleneck or a contraction event at some point in time. There was only one exception where a persistence (expansion) model was selected (Figure 5; Tables S6 and S7).

## 4 Discussion

Our comparative population genetic and demographic analyses, using genome‐wide SNP data and a system of psammophilous (dynamic coastal sand dunes) versus geophilous (stable compact soil habitat) demes of *Eutagenia* darkling beetles in the Cyclades archipelago, identified striking differences between the lineages inhabiting the two contrasting habitat types. First, despite strong and shared dispersal barriers, the geographic structuring of genetic variation is weaker and the inferred levels of inter‐island gene flow are higher among the psammophilous demes, compared to their geophilous counterparts. Second, despite the estimated higher levels of gene flow, we observed comparatively lower local genetic diversity and higher population differentiation levels in the psammophilous demes, coupled with a prevalence of bottleneck events in the inferred demographic models. These results are compatible with higher levels of local population extinction and/or more frequent founder events in the psammophilous demes, indicating that interdemic migration among the dynamic habitat patches might follow an extinction–recolonisation model. Third, the aforementioned intra‐ and inter‐demic genetic patterns observed in the psammophilous demes are surprisingly uniform across islands of different palaeogeographical history and levels of connectivity. On the contrary, the inferred patterns for the geophilous demes vary widely among islands in a predictable way (i.e., higher levels of gene flow among islands that have been connected more recently), thus they are clearly influenced by the geological dynamics that shaped the Cyclades archipelago. Our results are in line with the predictions of the HC hypothesis, while they extend its original focus on the evolution of dispersal traits by highlighting a wider range of mechanisms that can potentially drive distinct evolutionary dynamics between stable versus dynamic habitat specialists.

### 4.1 Same Dispersal Barriers but Contrasting Gene Flow Patterns

Under the HC hypothesis, stable habitat specialist taxa are expected to display relatively limited inter‐patch movement, leading to increased interdemic genetic divergence and more prominent geographic structure in comparison to dynamic habitat specialist taxa (Ribera 2008). Here, we find that the geophilous *Eutagenia* demes are grouped into well‐defined genetic clusters that correspond either to single islands or to groups of neighbouring islands with recent shared history (Figure 3). Accordingly, the majority of geophilous inter‐island deme pairs have diverged in isolation (i.e., without gene flow; Figure 4), while models involving continuous or contemporary migration are only selected for intra‐island deme pairs or between neighbouring islands that have been connected by long‐lasting and/or wide land bridges (Figure 4, Tables S8 and S9). On the contrary, and across the same geographic space, the genetic structuring of psammophilous demes is best explained by fewer, more widespread and less isolated clusters (Figure 3). At the same time, the inferred psammophilous clusters correspond weakly to the underlying (palaeo)geography of the study area, while there is archipelago‐wide gene flow across demes, regardless of the spatial configuration of interdemic dispersal barriers (Figure 4).

These results are in agreement with the expectation of increased dispersal propensity in dynamic habitat types. Considering the dispersal limitations of the studied taxa, which are apterous soil‐dwelling beetles, the high inter‐island dispersal propensity of the dynamic habitat specialists appears unintuitive. Similar results in previously studied systems were typically linked with some dispersal‐related trait, which seems to be absent from *Eutagenia*, given the morphological uniformity of both habitat specialist taxa. In the widely cited freshwater beetle examples, lentic species can have higher wing loading (body mass/wing area) and wing aspect ratio (wing length/wing width) compared to their sister species that specialise in lotic habitats (e.g., Arribas et al. 2012). Similarly, wolf spiders in coastal sand dunes can present a great variety of dispersal behaviours, from ballooning to swimming and diving, which have been correlated with the unpredictability of their habitat (Postiglioni et al. 2019 and references therein). To our knowledge, there are no reports of similar dispersal‐promoting traits for sand‐obligate darkling beetles, despite their numerous adaptations to coastal dune environments (see Fattorini 2023). On the contrary, the widespread loss of wings among psammophilous taxa has been suggested as a mechanism to avoid the costs of dispersal (Fattorini 2023) due to the risk of displacement from highly exposed patches.

While we cannot exclude the possibility of an unknown behavioural or physiological trait promoting dispersal in *Eutagenia*, the small body size of these beetles suggests a primary role of passive dispersal (e.g., Holzapfel and Harrell 1968; Kirchner, Krätzner, and Welter‐Schultes 1997; Osborne, Lomolino, and Rundell 2024). Passive inter‐island dispersal of flightless darkling beetles across the Aegean has been previously postulated and attributed to sea currents (e.g., Fattorini 2002; but see Sfenthourakis and Triantis 2017, for limitations of the former study) or wind and sand movement (e.g., Papadopoulou et al. 2009). Indeed, it has been suggested that beetles can have high seawater tolerance, pointing to ‘floating dispersal’ and ‘rafting’ as widespread mechanisms of island colonisation (Claridge et al. 2017; Peck 1994; Renault 2011; Ueno, Kitagawa, and Matsubayashi 2020). Similarly, a study of invertebrate populations in ephemeral freshwater rock pools has identified wind dispersal of egg propagules to play a crucial role in inter‐patch gene flow and recolonisation (Vanschoenwinkel et al. 2008). If passive dispersal is indeed the main mechanism that maintains inter‐island gene flow in *Eutagenia*, why do we see such marked differences between the two habitat types? The coastal sandy habitats presumably provide increased opportunities for dispersal over seawater via floating and rafting, while wind currents and aeolian sand transport could carry egg propagules or even adult beetles over long distances. This is not expected to happen as frequently in inland compact soil habitats, that is, the lack of disturbance by wind and sea currents in those habitats provides less opportunities for passive dispersal. Thus, by focusing on dispersal‐limited taxa, we have here demonstrated that the observed contrasting patterns of gene flow between stable and dynamic habitat types might not be necessarily linked to an adaptive organismal trait but it could be alternatively associated with the physical properties of the habitats themselves, which may differ in their potential to promote passive dispersal.

### 4.2 Local Extinction as a Major Constraint in Dynamic Habitats

The HC hypothesis predicts that increased gene flow in dynamic habitats will act against genetic drift as a homogenising force reducing inter‐patch differentiation (Ribera 2008). While this may be true for actively dispersed organisms, the inability of dispersal‐limited taxa to actively escape deteriorating conditions and patch destruction could amplify the risk of deme extirpation (e.g., Casagrandi and Gatto 2002; Walker and Gilbert 2023) in dynamic habitat patches, generating genetic signatures (i.e., genetic drift and reduced levels of genetic diversity) different from those originally proposed under the HC hypothesis. The strikingly lower genetic diversity and the higher genetic differentiation patterns observed in the psammophilous *Eutagenia* demes in comparison to their geophilous counterparts (Figure 2) are indeed not compatible with higher gene flow levels in the sandy habitats if the effects of local extinction and recolonisation are neglected.

Local population extinction and recolonisation are considered another form of gene flow (Slatkin 1985) that fits better with the observed genetic patterns in the psammophilous demes. In systems with high population turnover, when extinction rates are greater or equal to migration rates, local genetic diversity is expected to decrease (see Pannell 2003; Pannell and Charlesworth 1999). Inter‐population genetic differentiation can either increase or decrease depending on the mode of migration (i.e., ‘propagule‐pool’ migration where colonisers come from a single deme vs. ‘migrant‐pool’ migration, where colonisers come from multiple demes) and on the interplay among extinction rate, migration rate and the number of colonisers (see McCauley 1991; Wade and McCauley 1988). The high genetic differentiation among the psammophilous demes (Figure 2) and the inferred bottleneck events in the single‐deme models (Figure 5) point towards a ‘propagule pool’ migration model, although it is hard to distinguish between the two modes without explicit measurements of migration, as most natural populations fall in an intermediate category of the migration spectrum (McCauley 1991). In any case, our results suggest that local extinction imposes a major constraint for dispersal‐limited taxa associated with sandy coastal habitats, which overwrites the genetic signature of gene flow through passive dispersal.

The relatively high population turnover in the dynamic habitat patches can have important implications for the speciation patterns of the psammophilous dispersal‐limited taxa. Under a protracted speciation model (Etienne and Rosindell 2012; Rosindell et al. 2010), the locally isolated populations are considered as the main evolutionary units of diversification. In the psammophilous dispersal‐limited lineages, even if there are opportunities for local populations to become isolated and differentiate, the physical properties of the sandy coastal habitats are expected to cause frequent extirpation of the local populations under differentiation, thus hindering the successful completion of the speciation process (Harvey, Singhal, and Rabosky 2019). Given the stochastic nature of sandy patch destruction and the cyclical recurrence of sea‐level changes in a geological timescale, the psammophilous clade might be under a repeated evolutionary reset. Indeed, while the deep divergence of the western geophilous demes (Figures 3, S3, S4, S9B and S10) is compatible with a scenario of in situ speciation within Cyclades for the geophilous lineage, there is no indication for incipient speciation in the psammophilous lineage, which could be either attributed to more frequent local population extinction or increased inter‐island migration in the psammophilous clade (see Dynesius and Jansson 2013). Since our analyses demonstrated the key role of local population extinction in shaping the genetic variation and demographic history of the psammophilous demes, there is no reason to neglect its potential role in shaping the macroevolutionary patterns of the psammophilous clade too. Overall, our results suggest that local population extinction should be incorporated as another major component of the HC hypothesis that may underlie to an important extent the micro‐ and macroevolutionary patterns observed in dynamic habitat lineages, at least in the case of dispersal‐limited taxa.

### 4.3 The Asymmetry of Habitat Constraints

While empirical studies have often considered the expectations of the HC hypothesis as a hard dichotomy, in the original conceptualisation of the hypothesis, the authors reported outliers among the stable habitat taxa (Ribera and Vogler 2000), with wider distribution ranges than expected by the HC model. Such observations led to the idea that habitat constraints are asymmetrical, as increased dispersal propensity in dynamic habitats is tightly linked to long‐term lineage persistence (see Ribera 2008), while the dispersal rate of stable habitat taxa is not linked to lineage survival and thus it can vary more widely. We here provide support for such an asymmetry, by conducting side‐by‐side comparisons between dynamic versus stable habitat demes across a matrix of islands with varying levels of connectivity. We found uniform gene flow patterns for the psammophilous demes across islands irrespective of their palaeogeographical history and level of connectivity (see Figures 1 and 4), as expected under strict habitat constraints. If the constraints were equally stringent for the geophilous demes, we would expect similarly low levels of gene flow among islands, regardless of temporary dispersal opportunities, such as the formation of inter‐island land bridges. On the contrary, our demographic analyses inferred a wider range of models for geophilous demes (Figure 4) and even supported cases of contemporary or continuous migration between a few pairs of adjacent islands (e.g., between Naxos and Ano Koufonisi, Naxos and Mykonos, and Mykonos and Tinos; see also Tables S8 and S9), which formed long‐lasting connections and/or were connected via wide land bridges during the Pleistocene low sea‐level periods. Therefore, our results suggest that the dispersal propensity of the geophilous clade is primarily constrained by palaeography and island connectivity, rather than by habitat stability per se. Our findings enhance the assumption of Papadopoulou et al. (2009), who hypothesised that compact soil habitats do not intrinsically restrain dispersal, as they found winged geophilous beetles to present low inter‐island genetic differentiation across the Aegean archipelago.

The apparent relaxation of constraints in stable habitats is also supported by the results of previous phylogeographic studies on lotic taxa, which identified certain species with lower population differentiation (Lam, Toussaint, et al. 2018) or certain haplotypes with wider distributions (Rader, Unmack, and Moore 2017) than expected. Such exceptions point out the importance of controlling for organismal traits and historical/geographic factors when evaluating the HC hypothesis. Here, we confirm its predictability, by conducting comparisons between two co‐distributed lineages with distinct habitat associations but uniform morphological traits, while controlling for the connectivity history of the focal islands. Our findings suggest that the constraint asymmetry between dynamic and stable habitat taxa should be included more explicitly within the framework of the HC hypothesis and should be considered as a potential explanation for certain discrepancies identified by empirical studies (e.g., Murphy, Guzik, and Wilmer 2010; Short and Caterino 2009).

## 5 Conclusions

Our study finds broad support for the HC hypothesis, demonstrating that the durational stability of the habitat ‘templet’ imposes constraints on both population isolation and persistence. The durational stability of the focal habitat types was not measured directly and remains hypothetical, but the findings are fully consistent with the predictions of the HC hypothesis with respect to the role of habitat‐induced dispersal in driving micro‐ and macroevolutionary patterns. It may have been expected that organismal traits related to over‐sea dispersal propensity would be differentially selected between habitat types, but such traits were not evident in these flightless organisms. Instead, our study supports that the physical properties of the dynamic habitat determine the demographic parameters of populations. By focusing on a dispersal‐limited taxon, we also revealed the role of local population extinction as a major constraint for dynamic habitat specialists that can have a lasting imprint on the spatial structuring of genetic variation and even overwrite the genetic signature of gene flow. Further work is needed to assess if other passively dispersed taxa follow similar patterns and how other organismal traits (e.g., body size) may correlate with habitat‐induced dispersal and/or extinction–recolonisation dynamics. Given the key role of local population persistence under a protracted speciation model, the explicit inclusion of extinction–recolonisation dynamics within the habitat constraint model may provide additional insights into the links between the demography of habitat specialists and their macroevolutionary trajectories. Habitat durational stability could thus emerge as an important determinant of lineage survival and diversification, through its control of a subtle equilibrium between dispersal and extinction. This is especially relevant in a world where such equilibria are increasingly disturbed, as taxa with contrasting habitat preferences may also present differential responses to the impact of rapid environmental change.

## Author Contributions

E.M., A.P. and A.P.V. conceived the study. E.M., A.P. and I.A. organised the sampling scheme and performed the fieldwork. E.M. and A.P. performed laboratory work. E.M. conducted the analyses with help from J.O. E.M. wrote the manuscript with help from A.P. All authors read, revised and approved the manuscript.

## Conflicts of Interest

The authors declare no conflicts of interest.

## Benefit‐Sharing Statement

All collaborators who provided tissue samples are included as coauthors. Our data and results are available to the broader scientific community through the relevant public databases (as described in the Data Availability Statement).

## Supporting information


Appendix S1

Figures S1–S10

Tables S1–S10

## Data Availability

Raw Illumina reads per successfully sequenced individual have been deposited in the NCBI Sequence Read Archive (SRA), under BioProject PRJNA951918 (https://www.ncbi.nlm.nih.gov/bioproject/PRJNA951918), including the corresponding metadata files. All relevant files and scripts for data processing, as well as the input and auxiliary files of the presented analyses, have been made available via the DRYAD open‐access repository (https://doi.org/10.5061/dryad.bk3j9kdnc). Any supplementary methods, tables or figures that are referenced in the manuscript have been included as [Link], [Link], [Link]. All newly generated cytochrome *c* oxidase I (*cox1*) sequences that are included as part of Supporting Information analyses have been deposited in the NCBI Nucleotide database under accession numbers PQ435599–PQ435651.
