# Supplementary material for: Habitat Association Predicts Population Connectivity and Persistence in Flightless Beetles: A Population Genomics Approach Within a Dynamic Archipelago
Source: Mol Ecol. 2024 Nov 5;33(23):e17577. doi: 10.1111/mec.17577 (PMC11589695; doi:10.1111/mec.17577)
Supplement: Supplementary file 1 — Appendix S1 [file MEC-33-e17577-s002.pdf]

Supplementary File 1

Habitat association predicts population connectivity and persistence in flightless beetles: a population genomics approach within a dynamic archipelago

Emmanouil Meramveliotakis[1], Joaquín Ortego[2], Ioannis Anastasiou[3], Alfried P. Vogler[4,5], Anna Papadopoulou[1]

[1] *Department of Biological Sciences, Faculty of Pure and Applied Sciences, University of Cyprus, Nicosia, Cyprus*
[2] *Department of Ecology and Evolution, Estación Biológica de Doñana, EBD-CSIC, Seville, Spain*
[3] *Department of Biology, School of Science, National and Kapodistrian University of Athens, Athens, Greece*
[4] *Department of Life Sciences, Natural History Museum, London, United Kingdom*
[5] *Department of Life Sciences, Faculty of Natural Sciences, Silwood Park Campus, Imperial College London, Ascot, United Kingdom*

Corresponding author: Emmanouil Meramveliotakis, Department of Biological Sciences, Faculty of Pure and Applied Sciences, University of Cyprus, Nicosia, Cyprus; Email: emeram01@ucy.ac.cy

SINGLE LOCUS DELIMITATION OF PHYLOGENETIC CLUSTERS

1. METHODS

1.1. Taxon sampling and data generation

1.1.1. Cytochrome c oxidase I (*cox1*) mtDNA locus

A selection of 93 publicly available partial *cox1* sequences of *Eutagenia* spp. from the Aegean islands and neighboring mainlands (part of the Papadopoulou et al., 2008, 2009 datasets) were downloaded from the NCBI nucleotide database. An auxiliary set of 49 specimens were sampled and sequenced for the *cox1* locus, including representative specimens from the focal demes/islands (i.e., Cyclades), Rhodos (Dodecanese islands), Samos and Lesvos (North-East Aegean islands), Crete and the neighboring mainlands of Greece and Turkey. Sequences from 2 individuals of the genus *Dichillus* and 3 of the genus *Stenosis* were also included in the combined dataset as outgroup taxa. For the newly sampled specimens, total genomic DNA was extracted from the whole specimen, following a commercial bead-based protocol (Biosprint® 96 DNA Blood kit, Qiagen®) as implemented in the automated KingFisher Flex system (Thermo Fisher scientific). Fragments of *cox1* (829bp of the 3' end) were amplified, sequenced and edited following the protocols of Papadopoulou et al. (2008, 2009). The sequences were aligned using the global pair alignment method (G-INS-i) with 1000 iterations of refinement, as implemented in the MAFFT v7.520 (Katoh & Standley, 2013) software.

1.1.2. Muscular protein 20 (*Mp20*) nuclear locus

A selection of 87 publicly available *Mp20* partial sequences of *Eutagenia* spp. from the Aegean islands and neighboring mainlands (part of the Papadopoulou et al., 2009 dataset) were downloaded from the NCBI nucleotide database. Additionally, 3 sequences of *Stenosis*

spp. individuals were also included in the dataset as outgroup taxa. Multiple sequence alignment was performed using MAFFT v7.520, implementing the global pair alignment method (G-INS-i) with 1000 iterations of refinement.

1.2. Phylogenetic inference

Maximum likelihood (ML) gene trees were inferred separately for each locus, using IQ-TREE2 v2.3.6 (Minh et al., 2020). The appropriate model of nucleotide substitution and partitioning scheme was determined by MODELFINDER (Kalyaanamoorthy et al., 2017) as implemented in IQ-TREE2. For the *cox1* mtDNA locus, we allowed for the selection of a different model per codon site position, while for the *Mp20* nuclear locus the partitioning scheme by codon site position was only applied to each of the two exons and a single partition was defined for the intronic region. To infer the gene trees, each analysis was run with 10 independent replicates (value of stopping iteration was increased to 500 per replicate) and the best ML tree was selected. Branch support values were calculated using the ultrafast bootstrap approximation method (UFBoot; Hoang et al., 2018; Minh et al., 2013), with 1000 replicates.

1.3. Phylogenetic clusters delimitation with mPTP

Phylogenetic clusters were delimited on each of the two inferred gene trees, as implemented in mPTP v0.2.5 (Kapli et al., 2017). For each gene tree, the minimum branch length threshold was detected using the *--minbr_auto* option. Subsequently, genetic clusters were delimited using the multi-rate model (option *--multi*) after removing the outgroup taxa (option *--outgroup_crop*). Support for the delimitation schemes was evaluated using the Markov Chain Monte Carlo sampling method provided with the mPTP software. For each gene tree, 4 independent runs of 10,000,000 iterations each were conducted, with a sampling step of 1/1000 iterations and a burn-in fraction of 20%.

2. RESULTS

2.1. Single locus datasets

The *cox1* multiple sequence alignment included a total of 147 specimens (142 sequences of *Eutagenia* spp. and 5 sequences of outgroup taxa). The total alignment length was 829bp and included 269 parsimony informative sites (32%) and minimal missing data (0.7% total missing data, maximum observed value of 21% for one sequence). The *Mp20* alignment consisted of 90 sequences (87 sequences of *Eutagenia* spp. and 3 outgroup sequences). The total alignment length was 524bp and included two exons (positions 1 → 218 and 274 → 524) and one intron (positions 219 → 273). The alignment included 105 parsimony informative sites (20%) and a total amount of 6.4% missing data (a maximum of ~30% missing data was observed for two sequences).

2.2. Gene trees and phylogenetic clusters delimitation

The inferred ML gene trees and the results of the delimitation analyses for the partial *cox1* mtDNA and *Mp20* nuclear loci are presented in figures S11 and S12 respectively. For the *cox1* locus, the delimitation analysis identified 14 mtDNA lineages (i.e., mPTP clusters). The majority of them (13) were part of the geophilous clade, which presented strong geographic structure. On the contrary, all individuals in the psammophilous clade were grouped in a single mPTP cluster, despite the same sampling effort (i.e., the majority of islands are represented in both clades, with minimal exceptions, see Table S10 at the end of the document for the relevant information). For the *Mp20* nuclear locus, the delimitation analysis identified 3 large mPTP clusters. The geophilous clade was divided into two subclades (as well as mPTP clusters) corresponding to (a) the mainland of Turkey and Eastern Aegean islands, and (b) the mainland of Greece and the rest of Aegean islands, including some located in the Eastern Aegean. Similar to the *cox1* delimitation analysis, the psammophilous clade corresponds to a single mPTP cluster.

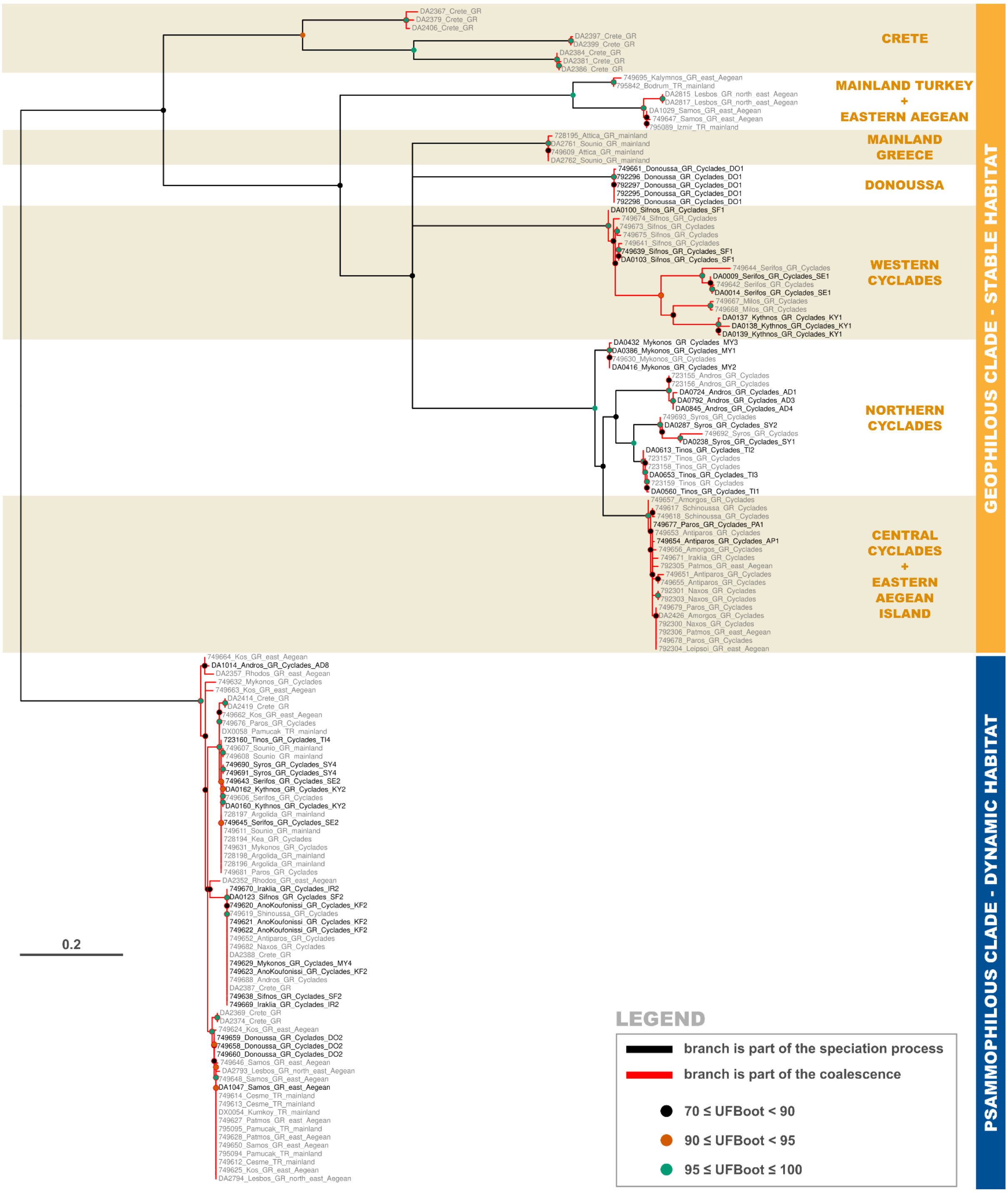

<u>Figure S11:</u> ML gene tree for the partial *cox1* mtDNA locus as inferred using IQ-TREE2. Outgroups have been pruned from the phylogeny and branches with UFBoot support lower than 70 have been collapsed. Tip labels with black colour denote specimens that are also part of the ddRADseq dataset (or at least sampled in the exact same location). For the geophilous clade (yellow bar) the geographic grouping of the different mtDNA lineages is also presented. The same was not feasible for the psammophilous clade, as there is no obvious geographic structure. The results of the delimitation analyses as implemented in mPTP are also represented on the tree with different branch colours as explained in the legend.

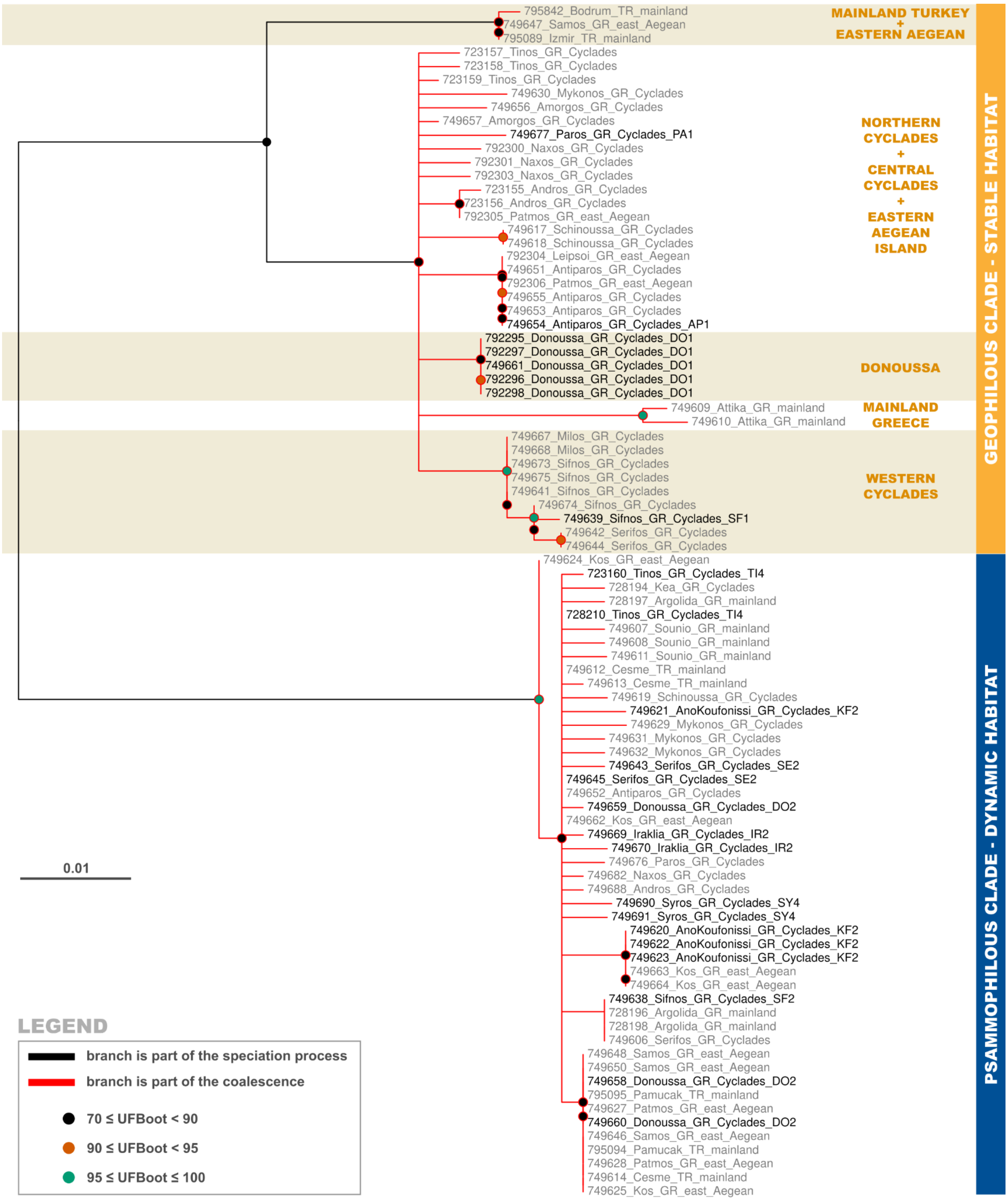

Figure S12: ML gene tree for the partial *Mp20* nuclear locus as inferred using IQ-TREE2. Outgroups have been pruned from the phylogeny and branches with UFBoot support lower than 70 have been collapsed. Tip labels with black colour denote specimens that are sampled in the exact same location as focal demes that are included in the ddRADseq dataset. For the geophilous clade (yellow bar) the geographic grouping of the different lineages is also presented. The same was not feasible for the psammophilous clade (blue bar), as there is no obvious geographic structure. The results of the delimitation analyses as implemented in mPTP are also represented on the tree with different branch colours as explained in the legend.

Table S11: Information on the specimens used in phylogenetic inference and delimitation analyses. For both datasets (i.e., *cox1* and *Mp20* loci) the NCBI database accession number of each specimen/sequence is given in the two rightmost columns. Cells with "*NA*" indicate that the corresponding sequence does not exist.

Specimen code	Genus	Lineage	Deme	Area	*cox1*	*Mp20*
DA2426_Amorgos_GR_Cyclades	*Eutagenia*	**geophilous**		Amorgos	PQ435643	*NA*
749656_Amorgos_GR_Cyclades	*Eutagenia*	**geophilous**		Amorgos	AM947737	FM877193
749657_Amorgos_GR_Cyclades	*Eutagenia*	**geophilous**		Amorgos	FM876525	FM877194
723155_Andros_GR_Cyclades	*Eutagenia*	**geophilous**		Andros	AM947686	FM877043
723156_Andros_GR_Cyclades	*Eutagenia*	**geophilous**		Andros	AM947687	FM877044
DA0724_Andros_GR_Cyclades_AD1	*Eutagenia*	**geophilous**	AD1	Andros	PQ435617	*NA*
DA0792_Andros_GR_Cyclades_AD3	*Eutagenia*	**geophilous**	AD3	Andros	PQ435618	*NA*
DA0845_Andros_GR_Cyclades_AD4	*Eutagenia*	**geophilous**	AD4	Andros	PQ435619	*NA*
749651_Antiparos_GR_Cyclades	*Eutagenia*	**geophilous**		Antiparos	AM947732	FM877188
749653_Antiparos_GR_Cyclades	*Eutagenia*	**geophilous**		Antiparos	AM947734	FM877190
749654_Antiparos_GR_Cyclades_AP1	*Eutagenia*	**geophilous**	AP1	Antiparos	AM947735	FM877191
749655_Antiparos_GR_Cyclades	*Eutagenia*	**geophilous**		Antiparos	AM947736	FM877192
728195_Attica_GR_mainland	*Eutagenia*	**geophilous**		Attica	FM876356	*NA*
749609_Attica_GR_mainland	*Eutagenia*	**geophilous**		Attica	FM876522	FM877148
749610_Attika_GR_mainland	*Eutagenia*	**geophilous**		Attika	*Low quality*	FM877149
795089_Izmir_TR_mainland	*Eutagenia*	**geophilous**		Izmir	AM947776	FM877346
795842_Bodrum_TR_mainland	*Eutagenia*	**geophilous**		Bodrum	AM947779	FM877400
DA2367_Crete_GR	*Eutagenia*	**geophilous**		Crete	PQ435629	*NA*
DA2379_Crete_GR	*Eutagenia*	**geophilous**		Crete	PQ435632	*NA*
DA2381_Crete_GR	*Eutagenia*	**geophilous**		Crete	PQ435633	*NA*
DA2384_Crete_GR	*Eutagenia*	**geophilous**		Crete	PQ435634	*NA*
DA2386_Crete_GR	*Eutagenia*	**geophilous**		Crete	PQ435635	*NA*
DA2397_Crete_GR	*Eutagenia*	**geophilous**		Crete	PQ435638	*NA*
DA2399_Crete_GR	*Eutagenia*	**geophilous**		Crete	PQ435639	*NA*
DA2406_Crete_GR	*Eutagenia*	**geophilous**		Crete	PQ435640	*NA*
749661_Donoussa_GR_Cyclades_DO1	*Eutagenia*	**geophilous**	DO1	Donoussa	FM876529	FM877198
792295_Donoussa_GR_Cyclades_DO1	*Eutagenia*	**geophilous**	DO1	Donoussa	FM876590	FM877274
792296_Donoussa_GR_Cyclades_DO1	*Eutagenia*	**geophilous**	DO1	Donoussa	FM876591	FM877275
792297_Donoussa_GR_Cyclades_DO1	*Eutagenia*	**geophilous**	DO1	Donoussa	FM876592	FM877276
792298_Donoussa_GR_Cyclades_DO1	*Eutagenia*	**geophilous**	DO1	Donoussa	FM876593	FM877277
749671_Iraklia_GR_Cyclades	*Eutagenia*	**geophilous**		Irakleia	AM947747	*NA*
749695_Kalymnos_GR_east_Aegean	*Eutagenia*	**geophilous**		Kalymnos	AM947767	*NA*
DA0137_Kythnos_GR_Cyclades_KY1	*Eutagenia*	**geophilous**	KY1	Kythnos	PQ435604	*NA*
DA0138_Kythnos_GR_Cyclades_KY1	*Eutagenia*	**geophilous**	KY1	Kythnos	PQ435605	*NA*
DA0139_Kythnos_GR_Cyclades_KY1	*Eutagenia*	**geophilous**	KY1	Kythnos	PQ435606	*NA*
792304_Leipsoi_GR_east_Aegean	*Eutagenia*	**geophilous**		Leipsoi	FM876594	FM877283
DA2815_Lesbos_GR_north_east_Aegean	*Eutagenia*	**geophilous**		Lesbos	PQ435648	*NA*
DA2817_Lesbos_GR_north_east_Aegean	*Eutagenia*	**geophilous**		Lesbos	PQ435649	*NA*
749667_Milos_GR_Cyclades	*Eutagenia*	**geophilous**		Milos	AM947743	FM877203
749668_Milos_GR_Cyclades	*Eutagenia*	**geophilous**		Milos	AM947744	FM877204
749630_Mykonos_GR_Cyclades	*Eutagenia*	**geophilous**		Mykonos	AM947712	FM877169
DA0386_Mykonos_GR_Cyclades_MY1	*Eutagenia*	**geophilous**	MY1	Mykonos	PQ435611	*NA*

DA0416_Mykonos_GR_Cyclades_MY2	*Eutagenia*	**geophilous**	MY2	Mykonos	PQ435612	*NA*
DA0432_Mykonos_GR_Cyclades_MY3	*Eutagenia*	**geophilous**	MY3	Mykonos	PQ435613	*NA*
792300_Naxos_GR_Cyclades	*Eutagenia*	**geophilous**		Naxos	AM947770	FM877279
792301_Naxos_GR_Cyclades	*Eutagenia*	**geophilous**		Naxos	AM947771	FM877280
792303_Naxos_GR_Cyclades	*Eutagenia*	**geophilous**		Naxos	AM947773	FM877282
749677_Paros_GR_Cyclades_PA1	*Eutagenia*	**geophilous**	PA1	Paros	AM947753	FM877212
749678_Paros_GR_Cyclades	*Eutagenia*	**geophilous**		Paros	AM947754	*NA*
749679_Paros_GR_Cyclades	*Eutagenia*	**geophilous**		Paros	AM947755	*NA*
792305_Patmos_GR_east_Aegean	*Eutagenia*	**geophilous**		Patmos	FM876595	FM877284
792306_Patmos_GR_east_Aegean	*Eutagenia*	**geophilous**		Patmos	FM876596	FM877285
749647_Samos_GR_east_Aegean	*Eutagenia*	**geophilous**		Samos	AM947729	FM877185
DA1029_Samos_GR_east_Aegean	*Eutagenia*	**geophilous**		Samos	PQ435625	*NA*
749642_Serifos_GR_Cyclades	*Eutagenia*	**geophilous**		Serifos	AM947724	FM877180
749644_Serifos_GR_Cyclades	*Eutagenia*	**geophilous**		Serifos	AM947726	FM877182
DA0009_Serifos_GR_Cyclades_SE1	*Eutagenia*	**geophilous**	SE1	Serifos	PQ435599	*NA*
DA0014_Serifos_GR_Cyclades_SE1	*Eutagenia*	**geophilous**	SE1	Serifos	PQ435600	*NA*
749617_Schinoussa_GR_Cyclades	*Eutagenia*	**geophilous**		Schinoussa	AM947699	FM877156
749618_Schinoussa_GR_Cyclades	*Eutagenia*	**geophilous**		Schinoussa	AM947700	FM877157
749639_Sifnos_GR_Cyclades_SF1	*Eutagenia*	**geophilous**	SF1	Sifnos	AM947721	FM877178
749641_Sifnos_GR_Cyclades	*Eutagenia*	**geophilous**		Sifnos	AM947723	FM877179
749673_Sifnos_GR_Cyclades	*Eutagenia*	**geophilous**		Sifnos	AM947749	FM877208
749675_Sifnos_GR_Cyclades	*Eutagenia*	**geophilous**		Sifnos	AM947751	FM877210
DA0100_Sifnos_GR_Cyclades_SF1	*Eutagenia*	**geophilous**	SF1	Sifnos	PQ435601	*NA*
DA0103_Sifnos_GR_Cyclades_SF1	*Eutagenia*	**geophilous**	SF1	Sifnos	PQ435602	*NA*
749674_Sifnos_GR_Cyclades	*Eutagenia*	**geophilous**		Sifnos	AM947750	FM877209
DA2761_Sounio_GR_mainland	*Eutagenia*	**geophilous**		Sounio	PQ435644	*NA*
DA2762_Sounio_GR_mainland	*Eutagenia*	**geophilous**		Sounio	PQ435645	*NA*
749692_Syros_GR_Cyclades	*Eutagenia*	**geophilous**		Syros	AM947765	*NA*
749693_Syros_GR_Cyclades	*Eutagenia*	**geophilous**		Syros	AM947766	*NA*
DA0238_Syros_GR_Cyclades_SY1	*Eutagenia*	**geophilous**	SY1	Syros	PQ435609	*NA*
DA0287_Syros_GR_Cyclades_SY2	*Eutagenia*	**geophilous**	SY2	Syros	PQ435610	*NA*
723157_Tinos_GR_Cyclades	*Eutagenia*	**geophilous**		Tinos	AM947688	FM877045
723158_Tinos_GR_Cyclades	*Eutagenia*	**geophilous**		Tinos	AM947689	FM877046
723159_Tinos_GR_Cyclades	*Eutagenia*	**geophilous**		Tinos	AM947690	FM877047
DA0560_Tinos_GR_Cyclades_TI1	*Eutagenia*	**geophilous**	TI1	Tinos	PQ435614	*NA*
DA0613_Tinos_GR_Cyclades_TI2	*Eutagenia*	**geophilous**	TI2	Tinos	PQ435615	*NA*
DA0653_Tinos_GR_Cyclades_TI3	*Eutagenia*	**geophilous**	TI3	Tinos	PQ435616	*NA*
749688_Andros_GR_Cyclades	*Eutagenia*	**psammophilous**		Andros	AM947761	FM877215
DA1014_Andros_GR_Cyclades_AD8	*Eutagenia*	**psammophilous**	AD8	Andros	PQ435620	*NA*
749620_AnoKoufonissi_GR_Cyclades_KF2	*Eutagenia*	**psammophilous**	KF2	AnoKoufonissi	AM947702	FM877159
749621_AnoKoufonissi_GR_Cyclades_KF2	*Eutagenia*	**psammophilous**	KF2	AnoKoufonissi	AM947703	FM877160
749622_AnoKoufonissi_GR_Cyclades_KF2	*Eutagenia*	**psammophilous**	KF2	AnoKoufonissi	AM947704	FM877161
749623_AnoKoufonissi_GR_Cyclades_KF2	*Eutagenia*	**psammophilous**	KF2	AnoKoufonissi	AM947705	FM877162
749652_Antiparos_GR_Cyclades	*Eutagenia*	**psammophilous**		Antiparos	AM947733	FM877189
728196_Argolida_GR_mainland	*Eutagenia*	**psammophilous**		Argolida	FM876357	FM877055
728197_Argolida_GR_mainland	*Eutagenia*	**psammophilous**		Argolida	FM876358	FM877056
728198_Argolida_GR_mainland	*Eutagenia*	**psammophilous**		Argolida	FM876359	FM877057

749612_Cesme_TR_mainland	*Eutagenia*	**psammophilous**		Cesme	AM947694	FM877151
749613_Cesme_TR_mainland	*Eutagenia*	**psammophilous**		Cesme	AM947695	FM877152
749614_Cesme_TR_mainland	*Eutagenia*	**psammophilous**		Cesme	AM947696	FM877153
DA2369_Crete_GR	*Eutagenia*	**psammophilous**		Crete	PQ435630	*NA*
DA2374_Crete_GR	*Eutagenia*	**psammophilous**		Crete	PQ435631	*NA*
DA2387_Crete_GR	*Eutagenia*	**psammophilous**		Crete	PQ435636	*NA*
DA2388_Crete_GR	*Eutagenia*	**psammophilous**		Crete	PQ435637	*NA*
DA2414_Crete_GR	*Eutagenia*	**psammophilous**		Crete	PQ435641	*NA*
DA2419_Crete_GR	*Eutagenia*	**psammophilous**		Crete	PQ435642	*NA*
749658_Donoussa_GR_Cyclades_DO2	*Eutagenia*	**psammophilous**	DO2	Donoussa	FM876526	FM877195
749659_Donoussa_GR_Cyclades_DO2	*Eutagenia*	**psammophilous**	DO2	Donoussa	FM876527	FM877196
749660_Donoussa_GR_Cyclades_DO2	*Eutagenia*	**psammophilous**	DO2	Donoussa	FM876528	FM877197
749669_Iraklia_GR_Cyclades_IR2	*Eutagenia*	**psammophilous**	IR2	Irakleia	AM947745	FM877205
749670_Iraklia_GR_Cyclades_IR2	*Eutagenia*	**psammophilous**	IR2	Irakleia	AM947746	FM877206
728194_Kea_GR_Cyclades	*Eutagenia*	**psammophilous**		Kea	AM947685	FM877054
749624_Kos_GR_east_Aegean	*Eutagenia*	**psammophilous**		Kos	AM947706	FM877163
749625_Kos_GR_east_Aegean	*Eutagenia*	**psammophilous**		Kos	AM947707	FM877164
749662_Kos_GR_east_Aegean	*Eutagenia*	**psammophilous**		Kos	AM947738	FM877199
749663_Kos_GR_east_Aegean	*Eutagenia*	**psammophilous**		Kos	AM947739	FM877200
749664_Kos_GR_east_Aegean	*Eutagenia*	**psammophilous**		Kos	AM947740	FM877201
DX0054_Kumkoy_TR_mainland	*Eutagenia*	**psammophilous**		Kumkoy	PQ435650	*NA*
DA0160_Kythnos_GR_Cyclades_KY2	*Eutagenia*	**psammophilous**	KY2	Kythnos	PQ435607	*NA*
DA0162_Kythnos_GR_Cyclades_KY2	*Eutagenia*	**psammophilous**	KY2	Kythnos	PQ435608	*NA*
DA2793_Lesbos_GR_north_east_Aegean	*Eutagenia*	**psammophilous**		Lesbos	PQ435646	*NA*
DA2794_Lesbos_GR_north_east_Aegean	*Eutagenia*	**psammophilous**		Lesbos	PQ435647	*NA*
749629_Mykonos_GR_Cyclades_MY4	*Eutagenia*	**psammophilous**	MY4	Mykonos	AM947711	FM877168
749631_Mykonos_GR_Cyclades	*Eutagenia*	**psammophilous**		Mykonos	AM947713	FM877170
749632_Mykonos_GR_Cyclades	*Eutagenia*	**psammophilous**		Mykonos	AM947714	FM877171
749682_Naxos_GR_Cyclades	*Eutagenia*	**psammophilous**		Naxos	AM947758	FM877213
DX0058_Pamucak_TR_mainland	*Eutagenia*	**psammophilous**		Pamucak	PQ435651	*NA*
795094_Pamucak_TR_mainland	*Eutagenia*	**psammophilous**		Pamucak	AM947777	FM877347
795095_Pamucak_TR_mainland	*Eutagenia*	**psammophilous**		Pamucak	AM947778	FM877348
749676_Paros_GR_Cyclades	*Eutagenia*	**psammophilous**		Paros	AM947752	FM877211
749681_Paros_GR_Cyclades	*Eutagenia*	**psammophilous**		Paros	AM947757	*NA*
749627_Patmos_GR_east_Aegean	*Eutagenia*	**psammophilous**		Patmos	AM947709	FM877166
749628_Patmos_GR_east_Aegean	*Eutagenia*	**psammophilous**		Patmos	AM947710	FM877167
DA2352_Rhodos_GR_east_Aegean	*Eutagenia*	**psammophilous**		Rhodos	PQ435627	*NA*
DA2357_Rhodos_GR_east_Aegean	*Eutagenia*	**psammophilous**		Rhodos	PQ435628	*NA*
749646_Samos_GR_east_Aegean	*Eutagenia*	**psammophilous**		Samos	AM947728	FM877184
749648_Samos_GR_east_Aegean	*Eutagenia*	**psammophilous**		Samos	AM947730	FM877186
749650_Samos_GR_east_Aegean	*Eutagenia*	**psammophilous**		Samos	AM947731	FM877187
DA1047_Samos_GR_east_Aegean	*Eutagenia*	**psammophilous**		Samos	PQ435626	*NA*
749606_Serifos_GR_Cyclades	*Eutagenia*	**psammophilous**		Serifos	AM947693	FM877145
749645_Serifos_GR_Cyclades_SE2	*Eutagenia*	**psammophilous**	SE2	Serifos	AM947727	FM877183
749643_Serifos_GR_Cyclades_SE2	*Eutagenia*	**psammophilous**	SE2	Serifos	AM947725	FM877181
749619_Schinoussa_GR_Cyclades	*Eutagenia*	**psammophilous**		Schinoussa	AM947701	FM877158
749638_Sifnos_GR_Cyclades_SF2	*Eutagenia*	**psammophilous**	SF2	Sifnos	AM947720	FM877177

DA0123_Sifnos_GR_Cyclades_SF2	*Eutagenia*	**psammophilous**	SF2	Sifnos	PQ435603	*NA*
749607_Sounio_GR_mainland	*Eutagenia*	**psammophilous**		Sounio	FM876520	FM877146
749608_Sounio_GR_mainland	*Eutagenia*	**psammophilous**		Sounio	FM876521	FM877147
749611_Sounio_GR_mainland	*Eutagenia*	**psammophilous**		Sounio	FM876524	FM877150
749690_Syros_GR_Cyclades_SY4	*Eutagenia*	**psammophilous**	SY4	Syros	AM947763	FM877216
749691_Syros_GR_Cyclades_SY4	*Eutagenia*	**psammophilous**	SY4	Syros	AM947764	FM877217
723160_Tinos_GR_Cyclades_TI4	*Eutagenia*	**psammophilous**		Tinos	AM947691	FM877048
728210_Tinos_GR_Cyclades_TI4	*Eutagenia*	**psammophilous**		Tinos	*NA*	FM877060
DA1024_Dichillus_sp	*Dichillus*	unknown		Syros	PQ435621	*NA*
DA1025_Dichillus_sp	*Dichillus*	unknown		Andros	PQ435622	*NA*
829595_Stenosis_syrensis	*Stenosis*	unknown		Naxos	FM876762	FM877418
DA1026_Stenosis_sp	*Stenosis*	unknown		Andros	PQ435623	*NA*
DA1027_Stenosis_sp	*Stenosis*	unknown		Andros	PQ435624	*NA*
829614_Stenosis_syrensis	*Stenosis*	unknown		Andros	*Not included*	FN545081
728163_Stenosis_sp	*Stenosis*	unknown		Peloponnese	*Not included*	FN545077

REFERENCES

Hoang, D. T., Chernomor, O., von Haeseler, A., Minh, B. Q., & Vinh, L. S. (2018). UFBoot2: Improving the Ultrafast Bootstrap Approximation. *Molecular Biology and Evolution*, *35*(2), 518–522. https://doi.org/10.1093/molbev/msx281

Kalyaanamoorthy, S., Minh, B. Q., Wong, T. K. F., von Haeseler, A., & Jermiin, L. S. (2017). ModelFinder: Fast model selection for accurate phylogenetic estimates. *Nature Methods*, *14*(6), 587–589. https://doi.org/10.1038/nmeth.4285

Kapli, P., Lutteropp, S., Zhang, J., Kobert, K., Pavlidis, P., Stamatakis, A., & Flouri, T. (2017). Multi-rate Poisson tree processes for single-locus species delimitation under maximum likelihood and Markov chain Monte Carlo. *Bioinformatics*, *33*(11), 1630–1638. https://doi.org/10.1093/bioinformatics/btx025

Katoh, K., & Standley, D. M. (2013). MAFFT Multiple Sequence Alignment Software Version 7: Improvements in Performance and Usability. *Molecular Biology and Evolution*, *30*(4), 772–780. https://doi.org/10.1093/molbev/mst010

Minh, B. Q., Nguyen, M. A. T., & von Haeseler, A. (2013). Ultrafast Approximation for Phylogenetic Bootstrap. *Molecular Biology and Evolution*, *30*(5), 1188–1195. https://doi.org/10.1093/molbev/mst024

Minh, B. Q., Schmidt, H. A., Chernomor, O., Schrempf, D., Woodhams, M. D., von Haeseler, A., & Lanfear, R. (2020). IQ-TREE 2: New Models and Efficient Methods for Phylogenetic Inference in the Genomic Era. *Molecular Biology and Evolution*, *37*(5), 1530–1534. https://doi.org/10.1093/molbev/msaa015

Papadopoulou, A., Anastasiou, I., Keskin, B., & Vogler, A. P. (2009). Comparative phylogeography of tenebrionid beetles in the Aegean archipelago: The effect of dispersal ability and habitat preference. *Molecular Ecology*, *18*(11), 2503–2517. https://doi.org/10.1111/j.1365-294X.2009.04207.x

Papadopoulou, A., Bergsten, J., Fujisawa, T., Monaghan, M. T., Barraclough, T., & Vogler, A. P. (2008). Speciation and DNA barcodes: Testing the effects of dispersal on the formation of discrete sequence clusters. *Philosophical Transactions of the Royal Society B: Biological Sciences*, *363*, 2987–2996. https://doi.org/10.1098/rstb.2008.0066
