# Supplementary material for: Habitat Association Predicts Population Connectivity and Persistence in Flightless Beetles: A Population Genomics Approach Within a Dynamic Archipelago
Source: Mol Ecol. 2024 Nov 5;33(23):e17577. doi: 10.1111/mec.17577 (PMC11589695; doi:10.1111/mec.17577)
Supplement: Supplementary file 2 — Figures S1–S10 [file MEC-33-e17577-s001.pdf]

Supplementary File 2 - Supplementary Figures

Habitat association predicts population connectivity and persistence in flightless beetles: a population genomics approach within a dynamic archipelago

Emmanouil Meramveliotakis[1], Joaquín Ortego[2], Ioannis Anastasiou[3], Alfried P. Vogler[4,5], Anna Papadopoulou[1]

[1] Department of Biological Sciences, Faculty of Pure and Applied Sciences, University of Cyprus, Nicosia, Cyprus
[2] Department of Ecology and Evolution, Estación Biológica de Doñana, EBD-CSIC, Seville, Spain
[3] Department of Biology, School of Science, National and Kapodistrian University of Athens, Athens, Greece
[4] Department of Life Sciences, Natural History Museum, London, United Kingdom
[5] Department of Life Sciences, Faculty of Natural Sciences, Silwood Park Campus, Imperial College London, Ascot, United Kingdom

Corresponding author: Emmanouil Meramveliotakis, Department of Biological Sciences, University of Cyprus, Nicosia, Cyprus; Email: emeram01@ucy.ac.cy

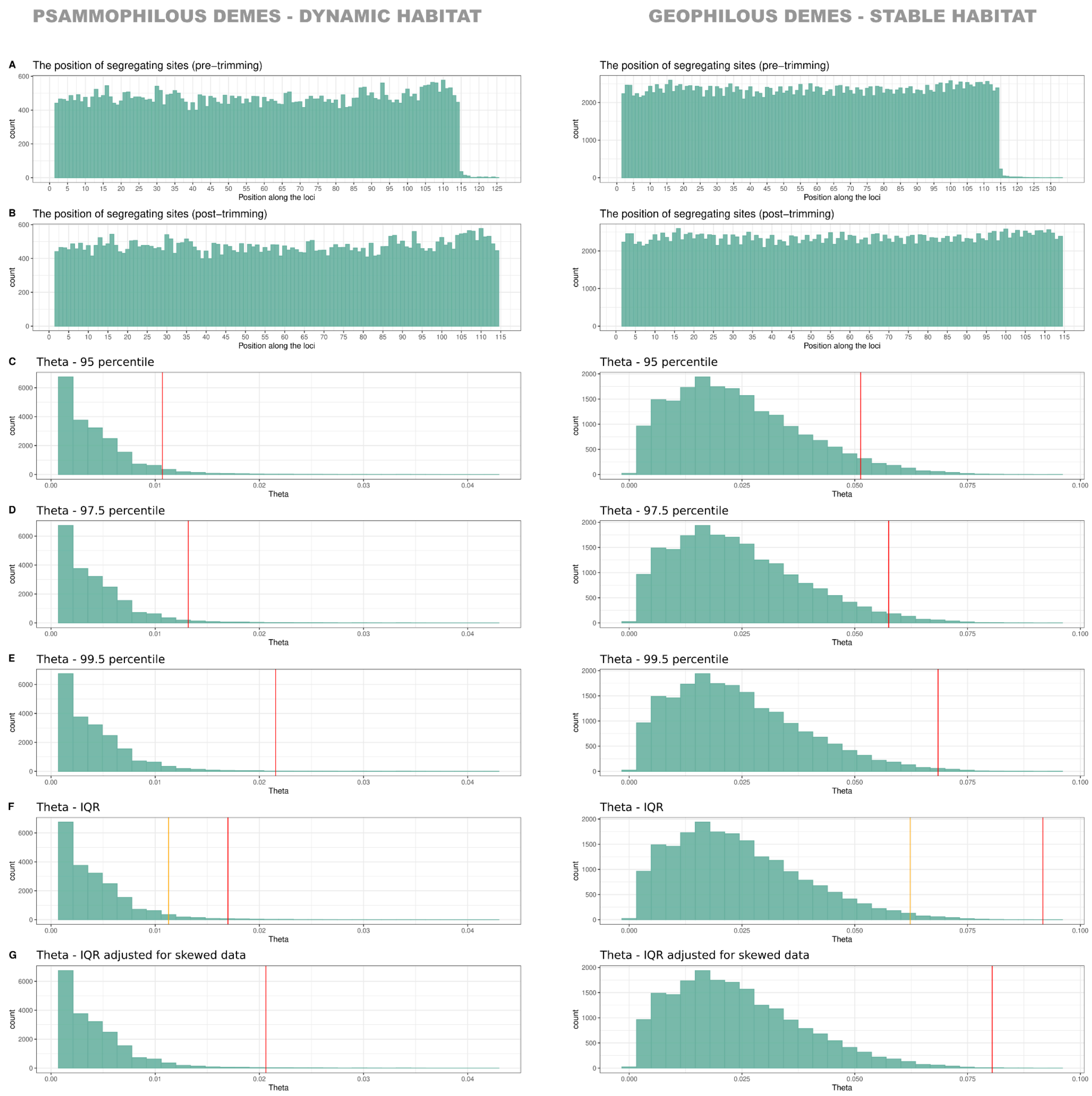

Figure S1: Filtering and trimming thresholds for the de novo assembly of the psammophilous and geophilous datasets. (A-B) Graphical representation of the position of segregating sites on the assembled loci before (A) and after (B) trimming. Trimming is needed as the difference in locus length creates a distribution “tail” with artificially low count of segregating sites. (C-G) Filtering thresholds (red and/or orange vertical lines) based on different criteria are depicted for both psammophilous and geophilous theta distributions. Panels C, D, and E depict thresholds based on different percentiles. Panels F and G depict thresholds based on the interquartile range and the Q3+1.5IQR / Q3+3IQR approach to detect outliers for normal and skewed distribution respectively (see Hubert & Van der Veeken, 2008, and Hubert & Vandervieren, 2008 for skewed distributions).

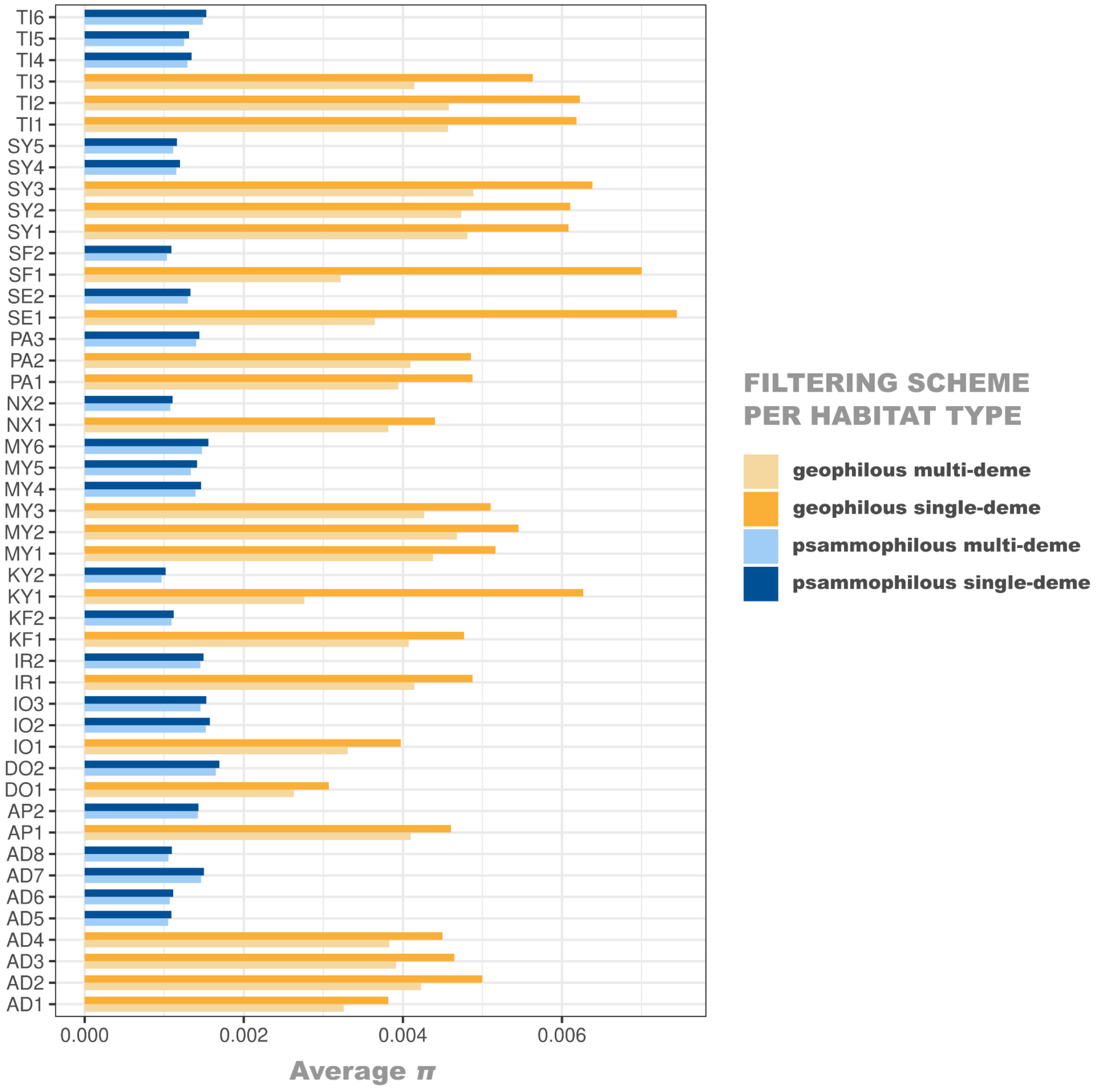

<u>Figure S2:</u> Average genetic diversity (π) per deme for different filtering schemes. Psammophilous demes are depicted with blue colour bars, while geophilous demes with yellow colour bars. Light hues denote a dataset-wide filtering scheme (i.e., variants are filtered based on their presence across all individuals), while darker hues denote deme-specific filtering (i.e., variants are filtered based on their presence across the individuals of each deme). For both filtering schemes a threshold of 50% presence is used.

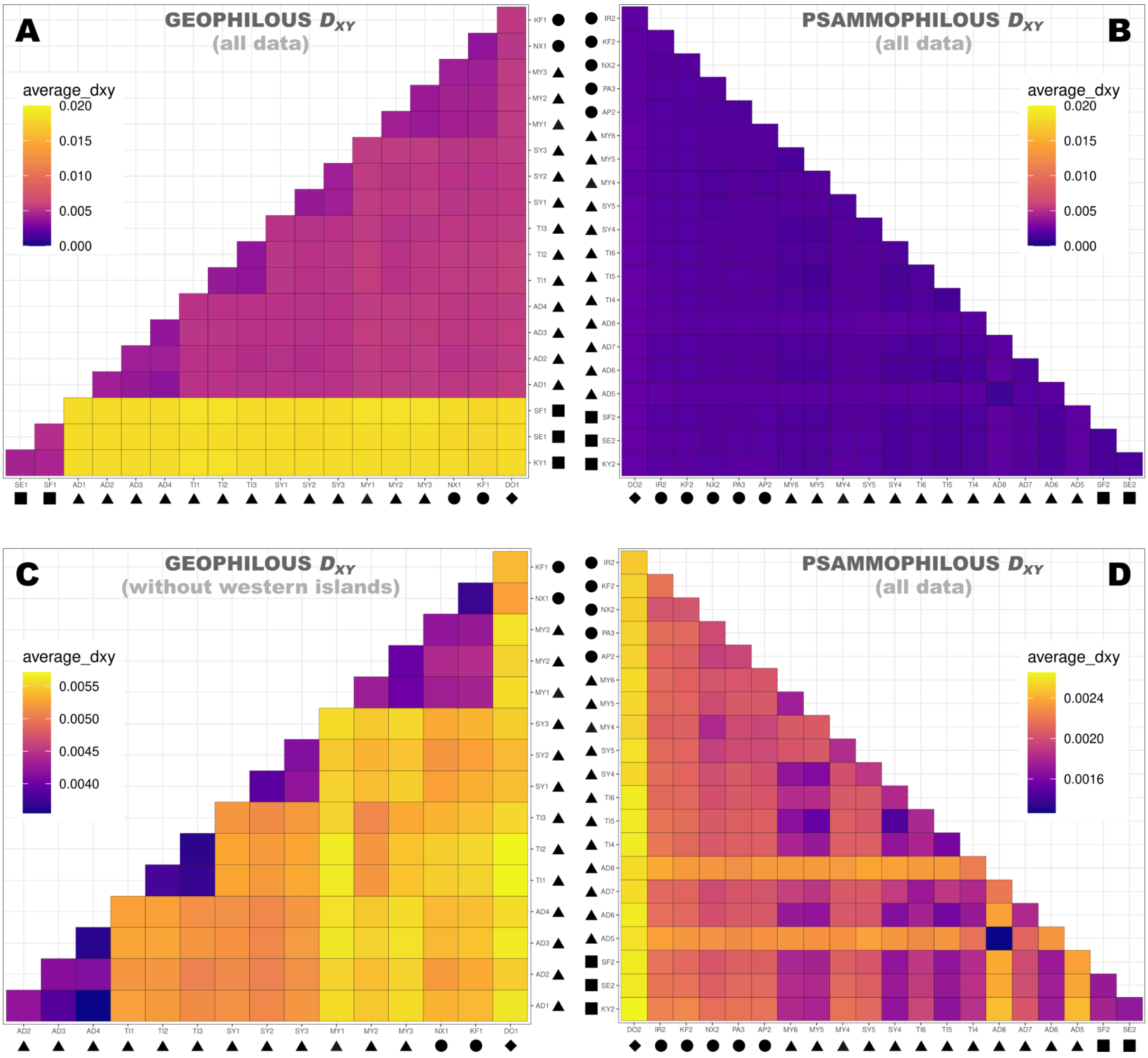

<u>Figure S3:</u> Heatmaps depicting average pairwise genetic divergence (D_{XY}) values between demes for each habitat type. The deme codes marked on the axes correspond to the respective islands: Andros (AD), Antiparos (AP), Donoussa (DO), Irakleia (IR), Ano Koufonisi (KF), Kythnos (KY), Mykonos (MY), Naxos (NX), Paros (PA), Serifos (SE), Sifnos (SF), Syros (SY), Tinos (TI). Palaeogeographic groups are marked by different shapes as follows: squares (■) represent western islands (i.e., Kythons, Serifos, Sifnos), triangles (▲) northern sector islands (i.e., Andros, Mykonos, Syros, Tinos), circles (●) southern sector islands (i.e., Ano Koufonisi, Antiparos, Irakleia, Naxos, Paros), and rhombuses (◆) the island of Donoussa. Only demes with 5 or more individuals are included. (A) Average D_{XY} values per geophilous deme pair. (B) Average D_{XY} values per psammophilous deme pair, with tile colours following the same scale as the geophilous heatmap, for comparison. (C) Average D_{XY} values per geophilous deme pair, excluding the western islands. (D) Average D_{XY} values per psammophilous deme pair, with tile colours scaled based on the psammophilous D_{XY} distribution.

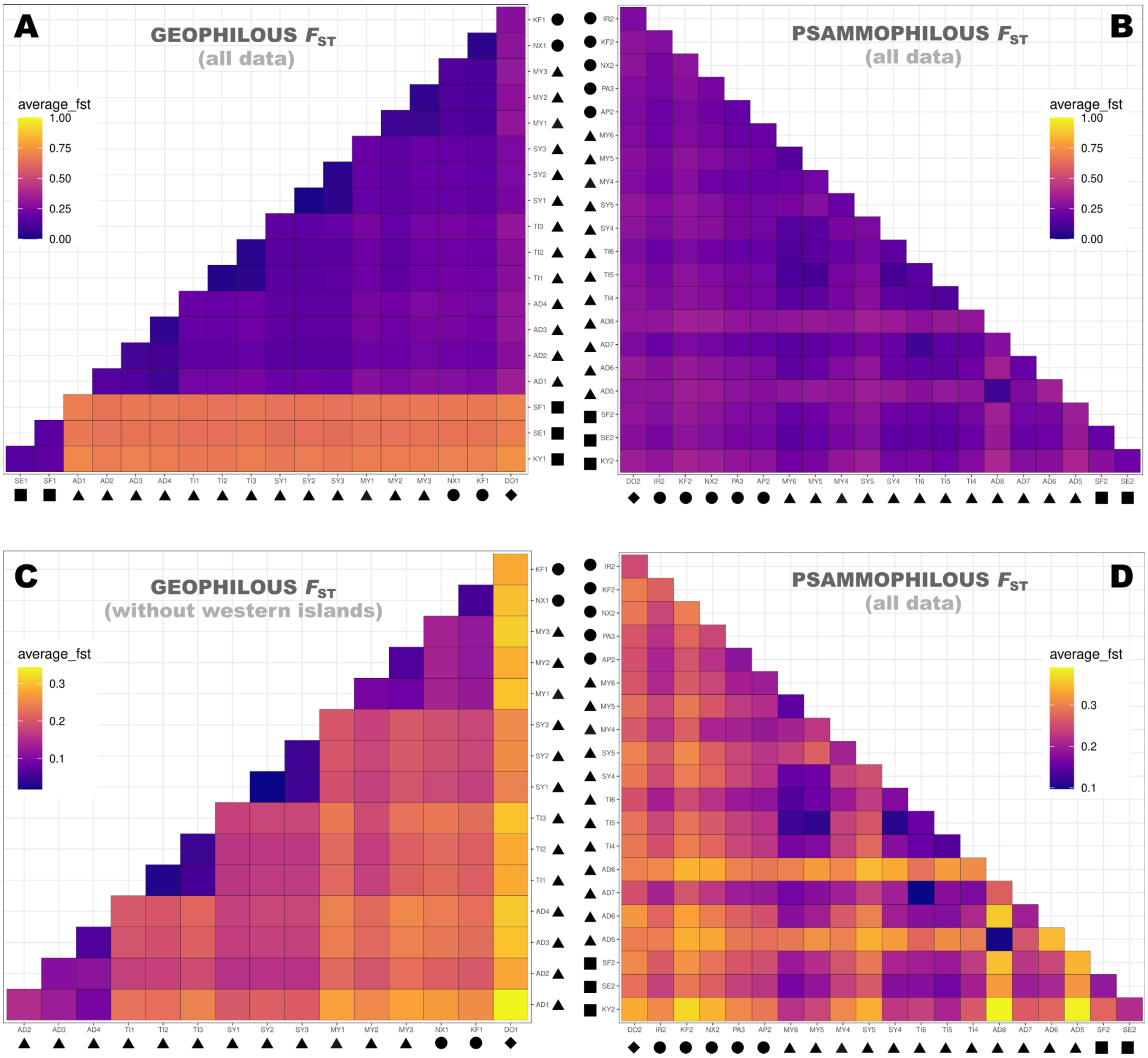

<u>Figure S4:</u> Heatmaps depicting average pairwise genetic differentiation (Hudson's F_{ST}) values between demes for each habitat type. The deme codes marked on the axes correspond to the respective islands: Andros (AD), Antiparos (AP), Donoussa (DO), Irakleia (IR), Ano Koufonisi (KF), Kythnos (KY), Mykonos (MY), Naxos (NX), Paros (PA), Serifos (SE), Sifnos (SF), Syros (SY), Tinos (TI). Palaeogeographic groups are marked by different shapes as follows: squares (■) represent western islands (i.e., Kythons, Serifos, Sifnos), triangles (▲) northern sector islands (i.e., Andros, Mykonos, Syros, Tinos), circles (●) southern sector islands (i.e., Ano Koufonisi, Antiparos, Irakleia, Naxos, Paros), and rhombuses (◆) the island of Donoussa. Only demes with 5 or more individuals are included. (A) Average F_{ST} values per geophilous deme pair. (B) Average F_{ST} values per psammophilous deme pair, with tile colours following the same scale as the geophilous heatmap, for comparison. (C) Average F_{ST} values per geophilous deme pair, excluding the western islands. (D) Average F_{ST} values per psammophilous deme pair, with tile colours scaled based on the psammophilous F_{ST} distribution.

PSAMMOPHILOUS DEMES - DYNAMIC HABITAT

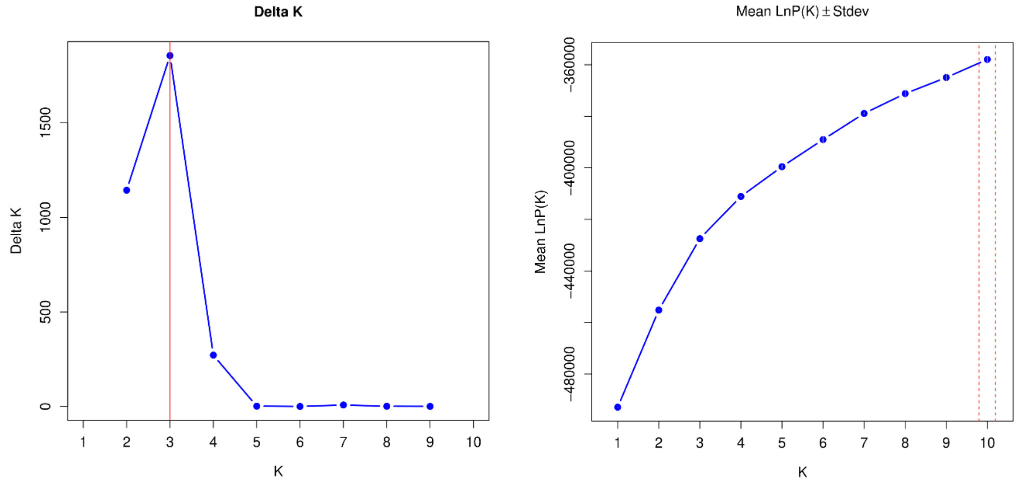

GEOPHILOUS DEMES - STABLE HABITAT - 1ST HIERARCHICAL LEVEL

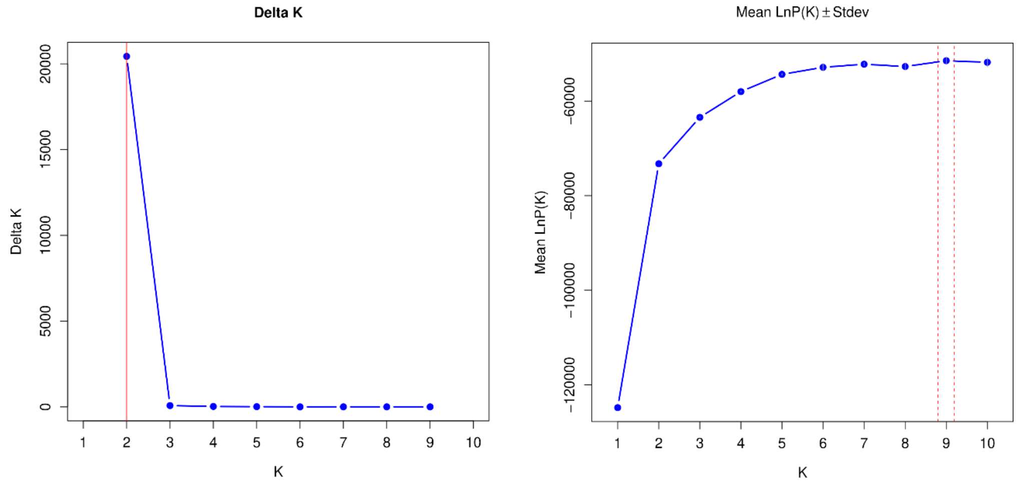

GEOPHILOUS DEMES - STABLE HABITAT - 2ND HIERARCHICAL LEVEL

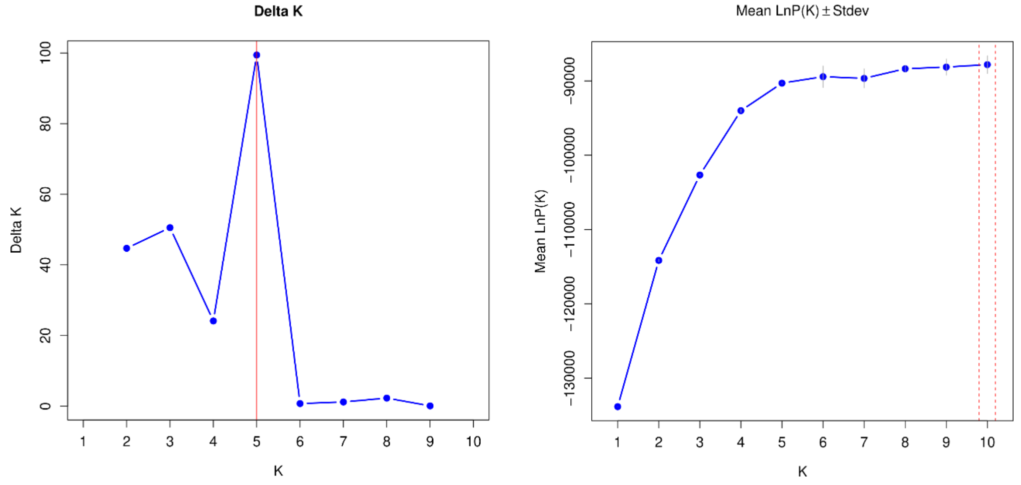

<u>**Figure S5:**</u> Δ*K* and mean log probability (LnP(*K*)) plots for 10 different *K* values as estimated in STRUCTURE v2.3.4. For the geophilous dataset, both hierarchical levels are depicted (i.e., all demes including the western islands (first hierarchical level - upper panel) and remaining demes excluding the western islands (second hierarchical level - bottom panel).

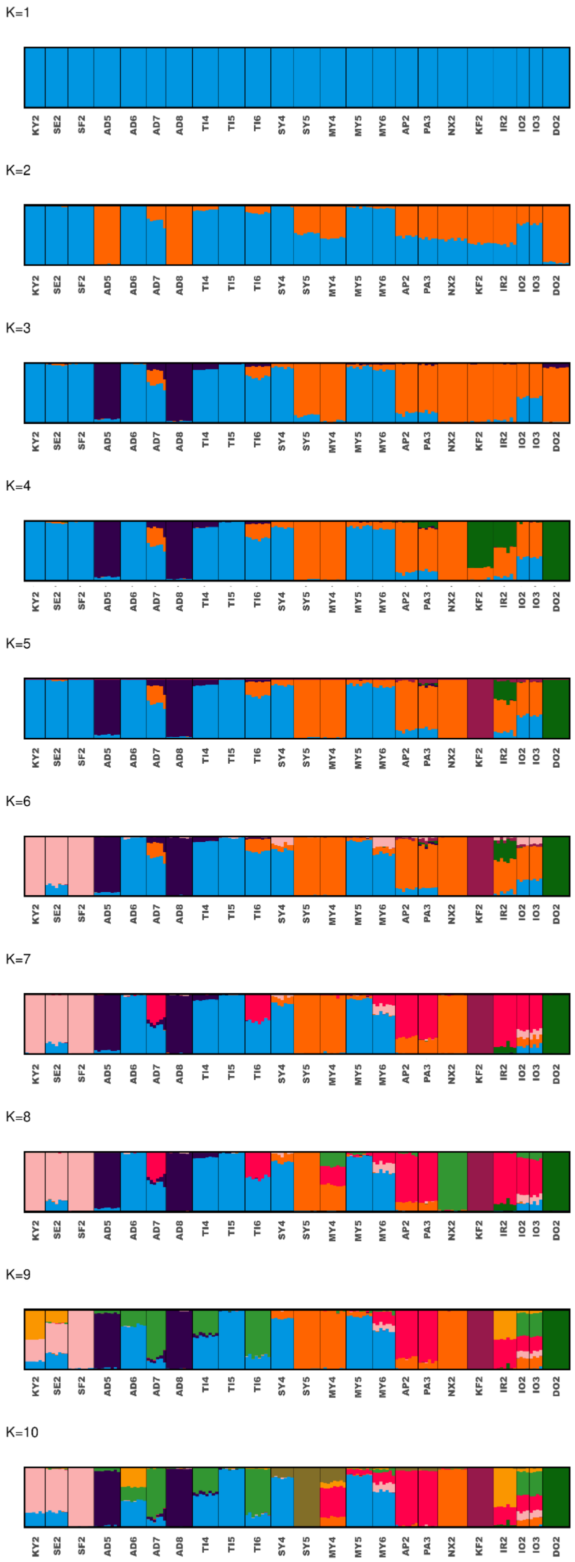

<u>Figure S6:</u> Results of genetic assignment for the psammophilous demes, across the different *K* values (1-10) as inferred in STRUCTURE v2.3.4.

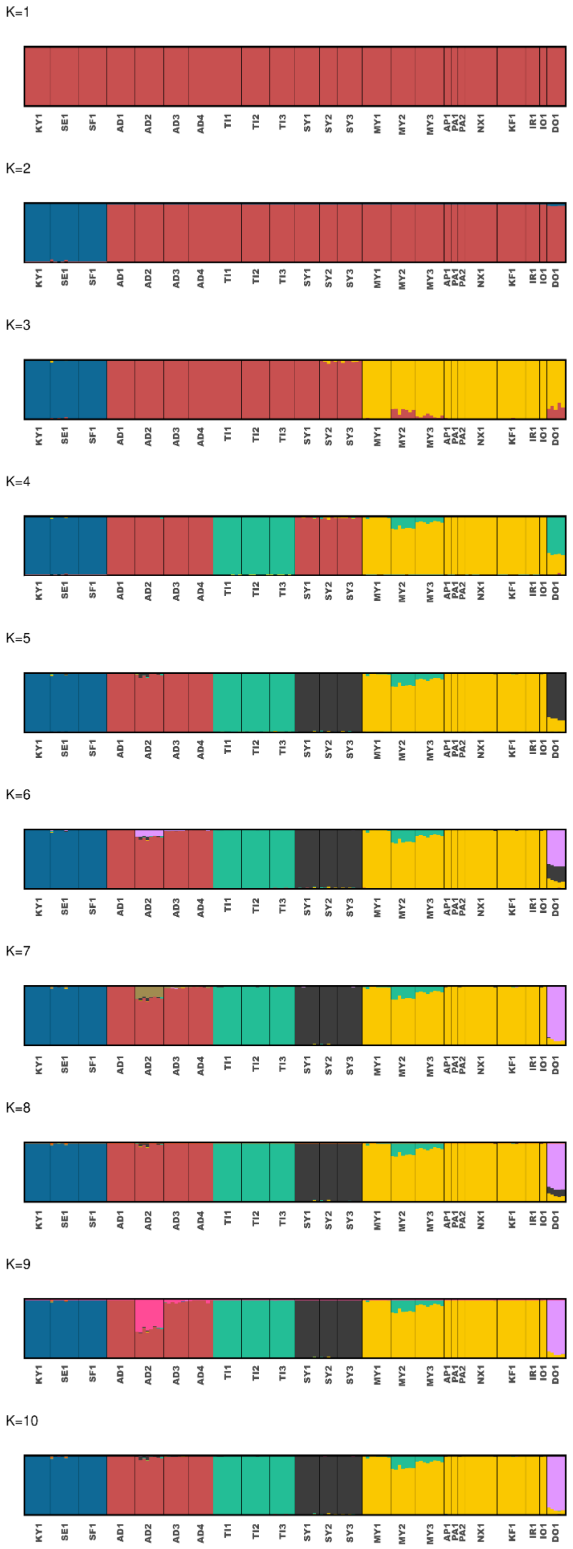

Figure S7: Results of genetic assignment for the first hierarchical level (i.e., all individuals across all islands) of geophilous demes, across the different *K* values (1-10) as inferred in STRUCTURE v2.3.4.

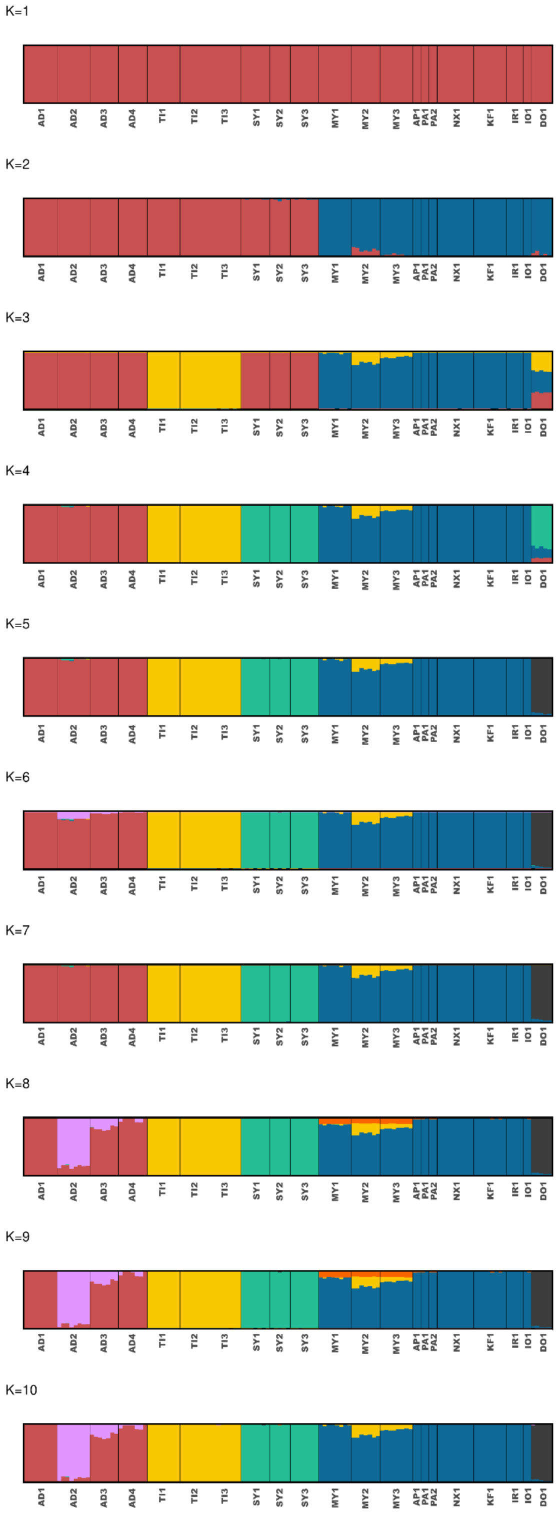

<u>Figure S8:</u> Results of genetic assignment for the second hierarchical level (i.e., after excluding individuals that were sampled across the western islands) of geophilous demes, across the different *K* values (1-10) as inferred in STRUCTURE v2.3.4.

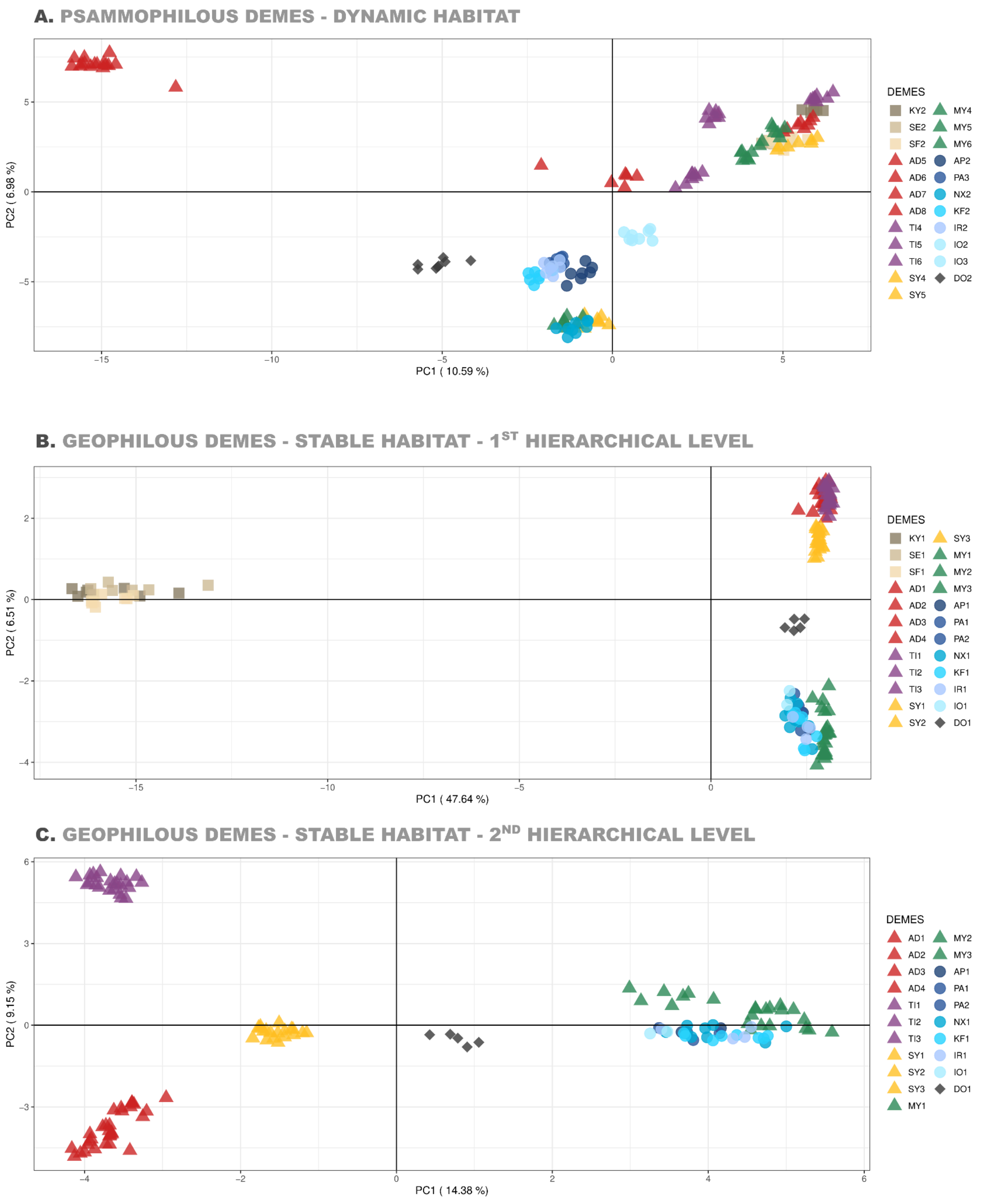

Figure S9: Principal component analyses (PCAs) of genetic variation for (A) the psammophilous demes (dynamic habitat), and (B,C) the two different hierarchical levels of clustering for the geophilous demes (stable habitat). Different islands are marked by different colours as indicated in the legend. Letters in deme codes correspond to the relevant islands: Andros (AD), Antiparos (AP), Donoussa (DO), Ios (IO), Irakleia (IR), Ano Koufonisi (KF), Kythnos (KY), Mykonos (MY), Naxos (NX), Paros (PA), Serifos (SE), Sifnos (SF), Syros (SY), Tinos (TI). Palaeogeographic groups are marked by different shapes as follows: squares (■) represent western islands (i.e., Kythons, Serifos, Sifnos), triangles (▲) northern sector islands (i.e., Andros, Mykonos, Syros, Tinos), circles (●) southern sector islands (i.e., Ano Koufonisi, Antiparos, Ios, Irakleia, Naxos, Paros), and rhombuses (◆) the island of Donoussa.

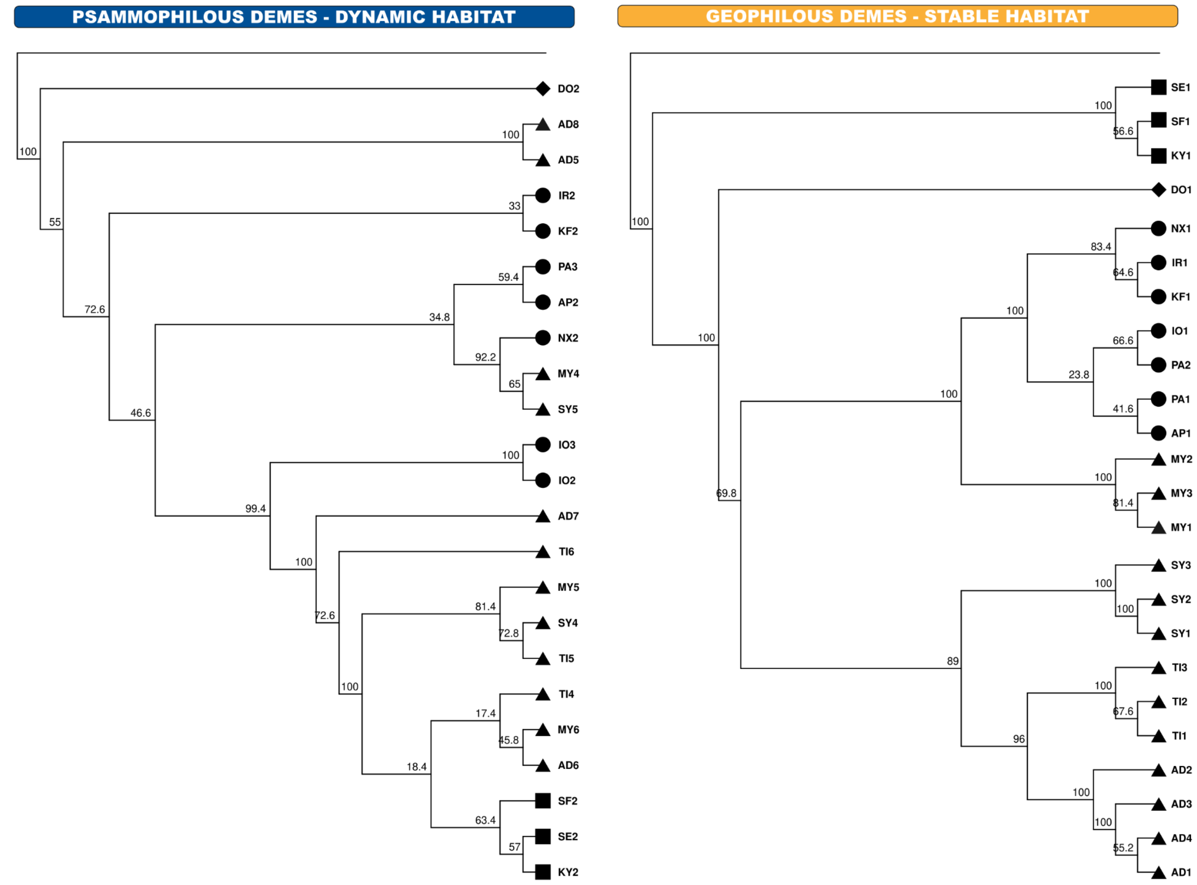

<u>Figure S10:</u> Population trees inferred using SVDQuartets. All possible quartets were evaluated using a total of 15,633 SNPs for the psammophilous populations (left) and 13,277 SNPs for the geophilous populations (right). Clade support values were estimated with 500 bootstrap replicates. Islands are coded as follows: AD (Andros), AP (Antiparos), DO (Donoussa), IO (Ios), IR (Irakleia), KF (Ano Koufonisi), KY (Kythnos), MY (Mykonos), NX (Naxos), PA (Paros), SE (Serifos), SF (Sifnos), SY (Syros), TI (Tinos). Terminal branches are marked based on the palaeogeographic grouping of the islands. Squares (■) represent western islands (i.e., Kythons, Serifos, Sifnos), triangles (▲) northern sector islands (i.e., Andros, Mykonos, Syros, Tinos), circles (●) southern sector islands (i.e., Ano Koufonisi, Antiparos, Ios, Irakleia, Naxos, Paros), and rhombuses (◆) the island of Donoussa.

REFERENCES

Hernangómez, D. (2023). *giscoR: Download Map Data from GISCO API - Eurostat* (0.4.0) [R]. https://zenodo.org/records/10055795

Hubert, M., & Van der Veeken, S. (2008). Outlier detection for skewed data. *Journal of Chemometrics*, *22*(3–4), 235–246. https://doi.org/10.1002/cem.1123

Hubert, M., & Vandervieren, E. (2008). An adjusted boxplot for skewed distributions. *Computational Statistics & Data Analysis*, *52*(12), 5186–5201. https://doi.org/10.1016/j.csda.2007.11.008

Pante, E., & Simon-Bouhet, B. (2013). marmap: A Package for Importing, Plotting and Analyzing Bathymetric and Topographic Data in R. *PLOS ONE*, *8*(9), e73051. https://doi.org/10.1371/journal.pone.0073051
